# Cell membrane-anchored anti-HIV single-chain antibodies and bifunctional inhibitors targeting the gp41 fusion protein: new strategies for HIV gene therapy

**DOI:** 10.1080/22221751.2021.2011616

**Published:** 2021-12-21

**Authors:** Yue Chen, Hongliang Jin, Xiaoran Tang, Li Li, Xiuzhu Geng, Yuanmei Zhu, Huihui Chong, Yuxian He

**Affiliations:** a NHC Key Laboratory of Systems Biology of Pathogens, Institute of Pathogen Biology, Chinese Academy of Medical Sciences and Peking Union Medical College, Beijing, People’s Republic of China; b Center for AIDS Research, Chinese Academy of Medical Sciences and Peking Union Medical College, Beijing, People’s Republic of China

**Keywords:** HIV, gene therapy, broadly neutralizing antibodies (bNAbs), fusion inhibitory peptide, glycosylphosphatidylinositol (GPI)

## Abstract

Emerging studies indicate that infusion of HIV-resistant cells could be an effective strategy to achieve a sterilizing or functional cure. We recently reported that glycosylphosphatidylinositol (GPI)-anchored nanobody or a fusion inhibitory peptide can render modified cells resistant to HIV-1 infection. In this study, we comprehensively characterized a panel of newly isolated HIV-1-neutralizing antibodies as GPI-anchored inhibitors. Fusion genes encoding the single-chain variable fragment (scFv) of 3BNC117, N6, PGT126, PGT128, 10E8, or 35O22 were constructed with a self-inactivating lentiviral vector, and they were efficiently expressed in the lipid raft sites of target cell membrane without affecting the expression of HIV-1 receptors (CD4, CCR5 and CXCR4). Significantly, transduced cells exhibited various degrees of resistance to cell-free HIV-1 infection and cell-associated HIV-1 transmission, as well as viral Env-mediated cell–cell fusion, with the cells modified by GPI-10E8 showing the most potent and broad anti-HIV activity. In mechanism, GPI-10E8 also interfered with the processing of viral Env in transduced cells and attenuated the infectivity of progeny viruses. By genetically linking 10E8 with a fusion inhibitor peptide, we subsequently designed a group of eight bifunctional constructs as cell membrane-based inhibitors, designated CMI01∼CMI08, which rendered cells completely resistant to HIV-1, HIV-2, and simian immunodeficiency virus (SIV). In human CD4^+^ T cells, GPI-10E8 and its bifunctional derivatives blocked both CCR5- and CXCR4-tropic HIV-1 isolates efficiently, and the modified cells displayed robust survival selection under HIV-1 infection. Therefore, our studies provide new strategies for generating HIV-resistant cells, which can be used alone or with other gene therapy approaches.

## Introduction

Over 37 million people are currently living with human Immunodeficiency virus (HIV), and about 1.8 million new infections per year continues to fuel the HIV pandemic. Antiretroviral therapy (ART) can efficiently suppress HIV replication in treated individuals and has dramatically reduced the morbidity and mortality associated with AIDS-related illness. Unfortunately, due to the establishment of a reservoir of quiescent, infected CD4^+^ T cells that harbour replication-competent virus, discontinuation of daily ART would lead to rapid viral rebound and continued disease progression, whereas lifelong treatment often results in cumulative toxicities and drug resistance [1,2]. Therefore, therapeutic interventions that can achieve a sterilizing or functional cure are among the top priorities of the HIV field.

The cure or functional cure of the “Berlin patient” and “London patient” through transplantation of allogeneic hematopoietic stem cells (HSCs) harbouring a naturally occurring mutation in the HIV coreceptor CCR5 (CCR5Δ32) suggests that infusion of HIV-resistant cells could be a viable treatment approach [3,4]. Currently, there are tremendous works to engineer CCR5 mutations into autologous cells using gene-editing technologies; however, the allogeneic transplantation of CCR5 gene-edited HSCs has so far achieved only very limited therapeutic efficacy in patients [5–7]. For example, Xu *et al*. recently reported successful transplantation and engraftment of allogeneic HSCs with CCR5 disruption by a CRISPR/Cas9 system in a patient with HIV-1 infection and acute lymphoblastic leukemia, which demonstrated safety but no therapeutic efficacy for HIV-1 was observed [8]. In addition to the potential of off-target CCR5 editing, there exist additional concerns over the CCR5Δ32 mutation. First, it only renders cells resistant to CCR5-tropic HIV strains; second, it can result in a shift of HIV tropism to CXCR4 usage [9]; and third, such a variant may increase susceptibility to some virus infections [10–12]. Thus, it is highly appreciated to develop alternative gene therapy approaches with improved safety and efficacy, and ideally, patient’s own cells are genetically engineered to simultaneously protect against HIV infection and promote immune reconstruction.

HIV entry into target cells is mediated by its envelope (Env) glycoprotein consisting of the surface subunit gp120 and transmembrane subunit gp41 [13]. While gp120 is responsible for binding with the primary cell receptor CD4 and a coreceptor (CCR5 or CXCR4), gp41 mediates fusion between viral and cell membranes, and both proteins induce neutralizing antibodies in HIV-infected individuals thus being major immunogens for vaccine development. Emerging studies demonstrate that membrane-anchored HIV entry inhibitors, such as gp41-derived fusion inhibitory peptides and broadly neutralizing antibodies (bNAbs), are particularly effective for generating HIV-resistant cells. In pilot studies, the fusion inhibitor peptides T20 and C46 were genetically engineered for cell surface expression through the membrane-spanning domain (MSD) of human low-affinity nerve growth factor receptor (LNGFR) or human tCD34 [14,15], while the fusion inhibitor peptide C34 was used to create resistant cells by genetically fusing it with the amino terminus of CCR5 and CXCR4 or with a glycosylphosphatidylinositol (GPI) attachment signal [16,17]. In humanized mice, CD4^+^ T cells expressing C34-CXCR4 were highly resistant to HIV-1 infection and had a selective survival advantage, and it has now been advanced to clinical trials [17,18]. Zhou and coworkers found that anti-HIV antibodies could render cells with various degrees of resistance to HIV-1 infection when genetically expressed on the cell surface as different formats through a GPI anchor [19–21]. Among several GPI-anchored constructs, the first-generation antibody X5, which targets the gp120 co-receptor-binding site, was found to endow cells with relatively higher resistance when tested against divergent HIV-1 strains [19]. In a humanized mouse model, GPI-X5-transduced primary CD4^+^ T cells were selected in the peripheral blood and lymphoid tissues upon HIV-1 infection and after being cotransfused with HIV-infected cells, the transduced CD4^+^ T cells could significantly reduce viral loads and viral RNA copy numbers [22]. In the recent years, a number of novel bNAbs have been identified from HIV-infected individuals, which target distinct neutralizing epitopes including the CD4-binding site (CD4bs), CD4-induced epitope (CD4i), V2- or V3-glycan site on gp120, and the membrane-proximal external region (MPER) on gp41, as well as the interface of the two subunits [23]. As reported, these newly isolated bNAbs possess dramatically improved anti-HIV activities in terms of their neutralizing potency and breadth, thus facilitating the design of HIV-1 vaccine in the concept of reverse vaccinology [24,25]. Definitely, the current generation bNAbs also offer promising candidates for developing therapeutic drugs and HIV-resistant cells.

Very recently, we demonstrated that GPI-anchored anti-gp120 nanobody m36.4 (GPI-m36.4) and fusion inhibitory peptide 2P23 (GPI-2P23) can efficiently modified cells with high levels of HIV resistance [26,27]. In this study, we focused to characterize a panel of new anti-HIV bNAbs as membrane-anchored inhibitors. In the panel, 3BNC117 and N6 recognize the CD4bs [28,29], PGT126 and PGT128 recognize the V3-glycan site [30,31], 35O22 recognizes the interface of gp120-gp41 on the trimeric envelope [32], and 10E8 targets the membrane-proximal external region (MPER) of gp41 [33]. The fusion genes encoding scFv formats of bNAbs were constructed by linking them with the GPI-anchoring sequence, and their cell surface expression and anti-HIV activities were comprehensively characterized from multiple angles. Among them, we identified that GPI-anchored 10E8 (GPI-10E8) could render target cells the most broad and potent resistance to divergent HIV-1 infections, viral Env-mediated cell–cell fusion, and cell-associated virion-mediated cell–cell transmission. In addition to blocking viral entry step, GPI-10E8 was characterized to have disruptive effect in HIV-1 Env and viral infectivity, verifying a multifaceted mode of action. In order to develop a more efficient HIV gene therapy, we then designed and characterized GPI-anchored bifunctional inhibitors by genetically linking 10E8 scFv with a 41-mer fusion inhibitor peptide P41, which conferred complete resistance to infections of HIV-1, HIV-2, and simian immunodeficiency virus (SIV). In human CD4^+^ T cells, GPI-10E8 and its bifunctional derivatives were capable of fully blocking infections of diverse HIV-1 strains regardless of their tropism and the modified cells displayed a robust selective survival advantage over unmodified cells following HIV-1 infection. Given a very high mutability with HIV, GPI-anchored inhibitors with a multifunctional activity have potential to overcome virus escape. Therefore, we think that the present studies provide novel strategies for generating HIV-resistant cells, which can be used alone or with other gene therapy approaches.

## Materials and methods

### Cell lines and plasmids

HEK293T cells were purchased from the American Type Culture Collection (ATCC; Rockville, MD). The human CD4^+^ T cell line CEM-SS expressing CCR5 (CEMss-CCR5) was a generous gift from Paul Zhou at the Institute Pasteur of Shanghai, Chinese Academy of Sciences, China. The plasmid encoding DSP_1-7_ and 293FT cells stably expressing CXCR4/CCR5/DSP_8–11_ were generous gifts from Zene Matsuda at the Institute of Medical Science of the University of Tokyo (Tokyo, Japan). TZM-bl cells stably expressing CD4/CCR5/CXCR4, molecular clones of HIV-1 (NL4-3, SG3.1, LAI.2, JR-CSF, MJ4, 89.6, THRO.c/2626, CH077.t/2627, and RHPA.c/2635) and of HIV-2 (ROD and ST), and plasmids encoding the “global panel” HIV-1 Envs as reference strains of subtypes A, B, C, G, A/C, A/E, and B/C that represent the worldwide HIV-1 epidemic were obtained through the AIDS Reagent Program, Division of AIDS, NIAID, NIH. Two plasmids encoding SIV Env (mac239 and smmPBj) were kindly provided by Jianqing Xu at the Shanghai Public Health Clinical Center, Fudan University, China.

### Construction of lentiviral vectors expressing transgenes

Recombinant lentiviral vectors expressing GPI-anchored transgenes were constructed as described previously [26,27]. In brief, fusion genes sequentially encoding scFv or a bispecific inhibitor, a His tag, and the C-terminal 34-amino acid GPI-anchoring signal sequence of decay accelerating factor (DAF) were synthesized and then cloned into the hPGK-driven self-inactivating lentiviral transfer vector pRRLsin.PPT.hPGK.WPRE between BamHI and SalI restriction sites. The bispecific transgenes were designed by linking the 10E8 scFv and a 41-mer fusion inhibitor peptide (P41) or its mutant in a tandem order of 10E8-(GGGGS)n-P41 or P41-(GGGGS)n-10E8 (n=1, 3, 5 or 7). To construct the vectors expressing a fusion gene with GFP, an internal 2A peptide signal was introduced and then inserted into a self-inactivating lentiviral transfer vector carrying a hEF1α promoter (pRRLsin.PPT.hEF1α.2A.GFP.WPRE) between the BamHI and SalI restriction sites.

### Production of recombinant lentiviruses carrying transgenes

Recombinant lentiviruses expressing transgenes were generated and titrated as described previously [26,27]. In brief, a total of 1.5 × 10^7^ HEK293T cells were seeded in a P-150 dish in 25 ml of complete DMEM and cultured at 37°C overnight. The cells were then cotransfected with 50 μg of lentiviral transfer vector encoding a fusion gene, 18.75 μg of packaging construct delta8.9 encoding Gag/Pol/Rev, and 7.5 μg of plasmid encoding vesicular stomatitis virus G envelope (VSV-G) by a linear polyethyleneimine (PEI) transfection reagent. After posttransfection 20 h, the culture medium was replaced with fresh complete DMEM containing 10% FBS and cells were cultured for an additional 24 h. Then, the virus-containing supernatants were harvested and centrifuged at 4000 rpm and 4 °C for 15 min, followed by filtration through a 0.45-μm filter. The lentiviral particles were then concentrated by ultracentrifugation and resuspended in RPMI1640 medium with 25mM HEPES and stored in aliquots in a −80°C freezer. Lentivirus titers were determined on HEK293T cells through the infection of various concentrations of lentiviruses in the presence of Polybrene (Sigma), and analyzed by the percentage of cells expressing His tag or GFP with a ﬂow cytometry (FACSCanto II; Becton, Dickinson, Mountain View, CA). The calculated titers were expressed as transducing units (TU) per milliliter.

### Generation of stable cell lines expressing GPI-anchored transgenes.

Target cells stably expressing GPI-anchored transgenes were generated as described previously [26,27]. Briefly, TZM-bl, 293FT, or CEMss-CCR5 cells were seeded in a 24-well plate at 5×10^4^ cells per well and incubated at 37°C overnight. The cells were transduced with 2 × 10^6^ TU of recombinant lentiviruses in the presence of 8 μg/ml polybrene (Sigma) and incubated for 24 h. The transduced cells were extensively washed and cultured in complete DMEM for 48 h. Then, the TZM-bl and 293FT cells expressing the transgenes were sorted using a mouse anti-His tag antibody (Invitrogen Life Technologies) and phycoerythrin (PE)-conjugated goat anti-mouse IgG antibody (eBioscience), and the CEMss-CCR5 cells expressing the transgenes were sorted by GFP expression.

### Cell surface expression of transgenes and HIV receptors determined by FACS analysis

The cell surface expression of GPI-anchored transgenes and HIV receptors was determined by flow cytometry analysis (FACS) as described previously [26,27]. To analyze the surface expression of GPI-scFv or a bifunctional inhibitor, transduced TZM-bl, 293FT, or CEMss-CCR5 cells were sequentially stained with a mouse anti-His tag antibody and a PE-conjugated goat anti-mouse IgG antibody or Alexa Fluor 647-conjugated goat anti-mouse IgG antibody (Invitrogen Life Technologies) for 60 min at 4°C. The stained cells were then washed twice with FACS buffer (phosphate-buffered saline [PBS] solution with 0.5% bovine serum albumin [BSA] and 2mM EDTA) and resuspended in 0.2 ml of FACS buffer containing 4% formaldehyde. FACS analysis was performed with a FACSCantoII instrument. To examine whether the expression of transgenes was truly linked with a GPI anchor, transduced TZM-bl cells were first treated with 5 U/ml phosphatidylinositol-specific phospholipase C (PI-PLC) (Invitrogen Life Technologies) in 0.5 ml 1×PBS, rocked for 30 min at 4°C, and then washed two times with FACS buffer to remove the remaining PI-PLC. Then, the cells were stained by antibodies and analyzed by FACS analysis as described above. To analyze if GPI-anchored transgenes affected the expression of CD4, CCR5, and CXCR4, transduced cells were stained with a PE-conjugated anti-human CD4, CD195, or CD184 antibody or an allophycocyanin-labeled anti-human CD4, CD195 or CD184 antibody (BD Bioscience) for 30 min at 4°C, washed twice with FACS buffer, and then fixed with 4% formaldehyde for FACS analysis.

### Analysis of transgene expression by Western blotting assay

To detect the expression of transgenes in transduced TZM-bl cells, 3×10^6^ cells were plated in a P-100 dish with 10 ml complete DMEM containing 10% FBS and incubated at 37°C in 5% CO_2_. After incubation for 24 h, the culture media were replaced with Freestyle 293 expression media (Invitrogen) and cultured for additional 48 h. Then, the cells and supernatants were harvested, respectively. Cells were lysed with ice-cold RIPA lysis buffer (25 mM Tris-HCl pH 7.6, 150 mM NaCl, 1% NP-40, 1% sodium deoxycholate, 0.1% SDS; Invitrogen) containing protease inhibitor cocktail (Roche). Proteins in supernatant were concentrated by ultrafiltration tube (15 ml, 10 kDa; Millipore). Samples were separated in 10% SDS-PAGE, transferred to a nitrocellulose membrane, followed by staining with mouse anti-His tag antibody (Sigma).

### Colocalization of GPI-anchored transgenes by confocal microscopy

Colocalization of GPI-anchored transgenes with a lipid raft marker (GM1) was determined by confocal microscopy as described previously [26,27]. Briefly, transduced TZM-bl cells were seeded in a 35-mm glass dish with a 14-mm bottom well (Cellvis) at 8,000 cells per well and incubated for 2 days at 37°C in 5% CO_2_. After two washes with PBS, the cells were fixed with 4% formaldehyde in PBS containing 1% BSA for 15 min and blocked with blocking buffer (5% goat serum in PBS containing 1% BSA) for 1 h. Subsequently, the cells were sequentially stained with a mouse anti-His tag antibody, Alexa Fluor 488- conjugated goat anti-mouse IgG antibody, and Alexa Fluor 555- conjugated CtxB (Invitrogen Life Technologies). The cells were washed with PBS three times and further stained with 4′,6-diamidino-2-phenylindole (DAPI) in permeabilization buffer (blocking buffer plus 0.5% saponin) for 7 min. Images were captured with a laser confocal microscope (Leica Microsystems, Wetzlar, Germany).

### Inhibition of GPI-anchored transgenes on infectious HIV-1/2 isolates

The inhibitory activity of GPI-anchored scFvs and bifunctional inhibitors on a panel of replication-competent HIV-1 (NL4-3, LAI.2, JRCSF, 89.6, THRO.c/2626, CH077.t/2627, RHPA.c/ 2635, and MJ4) and HIV-2 (ROD and ST) isolates were determined as described previously [26,27]. In brief, the viruses were produced by transfecting HEK293T cells (6 × 10^6^ cells in a P-100 mm dish) with 24 μg of plasmid encoding the molecular clone using a linear PEI transfection reagent. After posttransfection 48 h, the virus-containing supernatants were harvested and centrifuged at 2,000 × g for 5 min, passed through 0.45 μm filter to remove cell debris, and then stored in aliquots in a −80°C freezer. Viral titers were determined in TZM-bl cells by a 50% tissue culture infectious dose (TCID_50_) assay. To measure the inhibitory activity of GPI-anchored transgenes, 1×10^4^ of transduced TZM-bl cells in 96-well plate were inoculated with a virus at 200 TCID_50_ and incubated for 2 days at 37°C in 5% CO_2_. Then, the cells were lysed with lysis buffer, and luciferase activity was measured by a BrightGlo luciferase assay with a luminescence counter (Promega). To measure the inhibitory activity of transgenes in transduced CEMss-CCR5 cells, a virus at 1,000 TCID_50_ was added to cells (1 × 10^6^/well) and incubated overnight at 37 °C in 5% CO_2_. The infected cells were extensively washed with Hanks’ balanced salt solution (HBSS) and then cultured in complete DMEM. Intracellular HIV-1 p24 expression in infected cells was detected over time using PE-conjugated anti-p24 Gag antibody (Clone KC57, Beckman Coulter, Brea, CA) and analyzed together with GFP expression by FACS analysis.

### Inhibition of GPI-anchored transgenes on HIV-1 and SIV pseudoviruses

The inhibitory activity of GPI-anchored scFvs or bifunctional inhibitors against HIV-1 and SIV pseudoviruses was determined by a single-cycle infection assay as described previously [26,27]. The Env-pseudotyped viruses for the “global panel” of HIV-1 isolates, two SIV isolates (mac239 and smmPBj), and VSV-G were produced by cotransfection of HEK293T cells with a pSG3Δenv backbone plasmid and an Env-encoding plasmid using a linear PEI transfection reagent. Similar to the above, the pseudovirus-containing culture supernatants were harvested at 48 h posttransfection, centrifuged at 2,000 × g for 5 min, and passed through 0.45 μm filter, and then stored in aliquots in a −80°C freezer. Titers of pseudotyped virions were determined in TZM-bl cells by a TCID_50_ assay. To measure the inhibitory activity of transgenes in transduced TZM-bl cells, 200 TCID_50_ of a virus were added to the cells (1 × 10^4^ /well) and incubated at 37°C in 5% CO_2_ for 48 h. The cells were lysed and quantitated for luciferase expression as described above.

### Inhibition of GPI-anchored transgenes on HIV-1 env-mediated cell–cell fusion

The inhibitory activity of GPI-anchored scFvs or bifunctional inhibitors on Env-mediated cell–cell fusion was determined by a dual-split-protein (DSP)-based cell–cell fusion assay as described previously [26,27]. Briefly, 293FT cells stably expressing CCR5/CXCR4/DSP_8-11_ (refers to as 293FT_Target_) were first transduced with the lentiviral vectors encoding GPI-anchored transgenes. HEK293T cells (refers to as effector cells) were seeded in a 96-well plate at 1.5 × 10^4^ cells per well, incubated at 37°C with 5% CO_2_ overnight, and the cells were then cotransfected with a DSP_1-7_-expressing plasmid and an Env-expressing plasmid and incubated at 37°C for 24 h. Transgene- or mock-transduced 293FT_Target_ cells were resuspended in prewarmed culture medium and mixed with EnduRen live cell substrate (Promega), and then incubated at 37°C for 30 min. Next, the target cells (2.5 × 10^4^ /well) were added to effector cells, spun down to maximize cell–cell contact, and cocultured for 6 h. The fusion activity between the target and effector cells was measured by quantifying the production of the reporter luciferase as described above.

The inhibitory activity of GPI-anchored inhibitors on cell–cell fusion was also determined in transduced CEMss-CCR5 cells as described previously [21]. In brief, 1×10^5^ RHPA.c/2635-infected CEMss-CCR5 cells (donor cells) were mixed with 5×10^5^ vector-transduced CEMss-CCR5 cells (target cells), and seeded into a 12-well plate for coculturing. For comparison, equivalent donor cells and target cells were simultaneously co-cultured in a transwell chamber (12-well 0.4 mm polyester-membrane dishes, Corning Life Sciences, Corning, NY), in which RHPA.c/2635-infected donor cells were added to the transwell insert and transgene-expressing target cells were seeded into the bottom chamber. Cell–cell fusion was monitored after about 48 h of incubation, and images of syncytia were captured with a Nikon TE2000 epiﬂuorescence microscope running MetaMorph software (Molecular Devices, San Jose, USA). The numbers of syncytia (3 pictures per co-culture sample) were counted manually.

### Inhibition of GPI-anchored transgenes on cell–cell HIV-1 transmission

The inhibitory activity of GPI-anchored scFvs or bifunctional inhibitors on cell–cell HIV-1 transmission was determined as described previously [26,27]. In the assay, the infection of a R5-tropic virus in TZM-bl cells depends on polycationic supplements (DEAE-dextran) as cell-free virions but not during cell–cell transmission. TZM-bl cells were transduced with a GPI-anchored transgene as a target, and CEMss-CCR5 cells were infected with a CCR5-tropic virus (RHPA.c/2635, THRO.c/2626, or MJ4) as a donor. After donor cells and target cells were cocultured for 36 h, infection of TZM-bl cells was evaluated by quantifying the production of the luciferase reporter as described above.

### Effects of GPI-10E8 on HIV-1 Env processing and the infectivity of progeny viruses

The effects of GPI-10E8 along with GPI-FluIgG03 as a control on HIV-1 Env processing and the infectivity of progeny viruses were assayed as described previously [26]. In order to exclude the potential impacts caused by a lentiviral vector, the expression cassette of GPI-10E8 was also subcloned into the plasmid expression vector pcDNA3.1. HEK293T cells were cotransfected with 0.8 μg of pcDNA3.1-based vector and 0.8 μg of proviral clone of HIV-1 NL4-3 or transmitter/founder HIV-1 strain THRO.c/2626 by using a linear PEI transfection reagent. After cotransfection 12 h, the cell culture supernatants were replaced with fresh DMEM and incubated for an additional 36 h. The virus-containing supernatants were harvested and centrifuged at 4,000 rpm and 4°C for 15 min, followed by filtration with a 0.45-μm filter. To measure the infectivity of the progeny virions, the collected supernatants containing 0.25 ng of p24 antigen were added to TZM-bl cells (1 × 10^4^/well) and incubated for 2 days at 37 °C in 5% CO_2_. The cells were then lysed and quantitated for luciferase expression as described above.

The cotransfected cells were also used to analyze the effects of GPI-10E8 and GPI-FluIgG03 on the expression and processing of viral gp160 by Western blotting. Briefly, the cells were lysed by ice-cold RIPA buffer (Invitrogen) containing protease inhibitors (Roche) to generate proteins. The cell proteins were separated by SDS-PAGE and transferred to a nitrocellulose membrane, which was then blocked with a 5% nonfat dry milk solution in TBS-Tween 20 at room temperature for 1 h. The membrane was incubated overnight at 4°C with a rabbit anti-gp120 polyclonal antibody (SinoBiological, Beijing, China), the human anti-gp41 monoclonal antibody 10E8, or a mouse anti-*β* actin antibody (Sigma). After three washes with TBS-Tween 20, the membrane was incubated with IRDye 680LT goat-anti-rabbit IgG, IRDye 680LT goat-anti-mouse IgG, or IRDye 800CW goat-anti-human IgG for 2 h at room temperature. Images were scanned with an Odyssey infrared imaging system (LI-COR Biosciences, Lincoln, NE, USA).

### Cytotoxicity and anti-HIV activity of GPI-anchored bifunctional inhibitors in human primary CD4^+^ T cells

Human primary CD4^+^ T cells were enriched from peripheral blood mononuclear cells (PBMCs) by negative selection with CD4^+^ T Cell Isolation Kit (Miltenyi Biotec) and maintained in complete RPMI 1640 medium (Gibco) supplemented with 10% FBS (Gibco), 1% penicillin/streptomycin (HyClone) and 150 IU/ml human rIL-2 (Peprotech). CD4^+^ T cells were stimulated by mixing with anti-CD3/anti-CD28-coated magnetic beads (Gibco) at a bead-to-cell ratio of 1:1. After 24 h of stimulation, 2 × 10^5^ of CD4^+^ T cells were transduced with the CMI02 and CMI06 vectors at a multiplicity of infection (MOI) of 60, spinoculated at 2000 X g for 2 h at 32°C, and incubated at 37°C for 4 h. Then, the culture medium was replaced with fresh complete RPMI 1640 medium. 72 h after transduction, the cells were de-beaded and applied to detect the transduction efficiency by FACS analysis. Cell viability of transgene-transduced primary CD4^+^ T cells was measured using Cell Counting Kit-8 (Abbkine). Briefly, the cell concentration was adjusted to 3×10^5^/ml, and 100 μl of cell suspension were seeded to each well of a 96-well plate and then cultured at 37°C for 48 h. Subsequently, 10 µl of CCK-8 reagent were added to each well followed by incubation for 4 h. Absorbance values at the wavelength of 450 nm were then measured for each well using an ELISA plate reader (Thermo Fisher Scientific, Inc.) and cell viability (%) were calculated. The anti-HIV activity of transduced primary CD4^+^ T cells was measured as described above for transduced CEMss-CCR5 cells.

### Statistical analysis

GraphPad Prism 6 software was applied to statistically analyze data. One-way analysis of variance with Tukey’s multiple comparisons was used to identify significant differences among groups. A *P* value < 0.05 was considered significant.

## Results

### GPI-anchored bNAbs are efficiently expressed in the membrane lipid raft sites of transduced cells and do not affect the expression of HIV receptors

**Figure 1.**
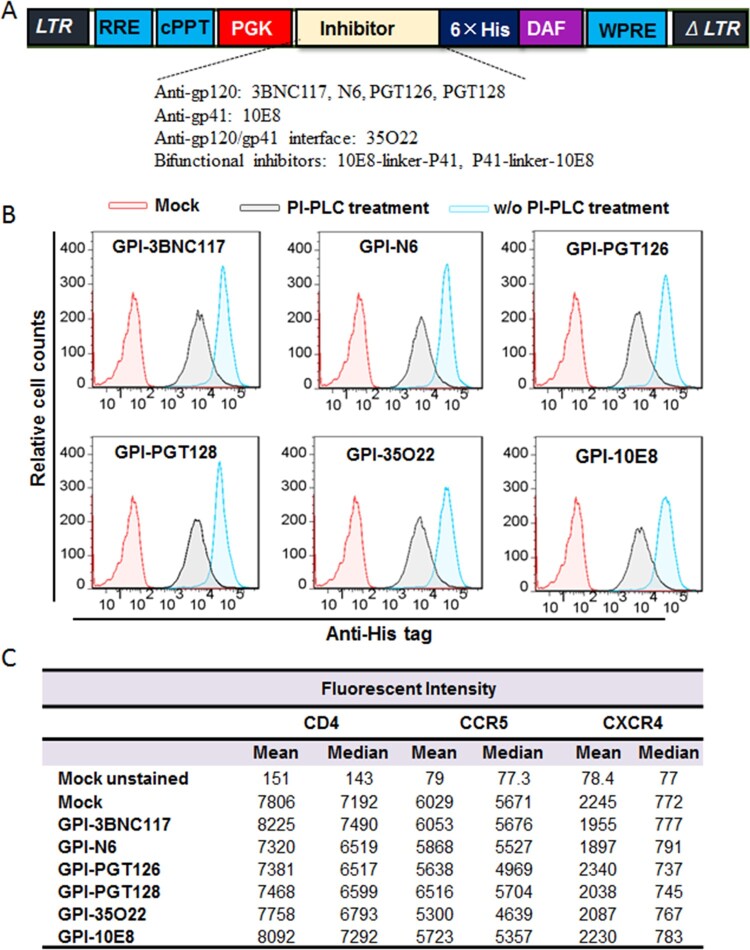
**Expression of GPI-scFvs in transduced TZM-bl cells and their effects on CD4, CCR5, and CXCR4. (A)** Schematic diagram of lentiviral transfer vector expressing a GPI-scFv or bifunctional inhibitor. The encoding sequence of scFv or a bifunctional inhibitor was genetically linked with the sequences encoding a His tag and the GPI attachment signal of DAF. LTR, long terminal repeat; RRE, Rev response element; cPPT, central polypurine track; PGK, human PGK promoter; DAF, delay-accelerating factor; WPRE, woodchuck hepatitits virus posttranscription regulatory element. **(B)** The expression of GPI-scFvs on the surface of transduced TZM-bl cells with or without PI-PLC treatment was determined by FACS analysis using an anti-His tag antibody. **(C)** Effects of GPI-scFvs on the expression of HIV receptors. The expression levels of CD4, CCR5, and CXCR4 on the surface of GPI-scFv-transduced TZM-bl cells were detected by FACS analysis using a PE-conjugated anti-human CD4, CCR5, or CXCR4 antibody and judged by the fluorescence intensity.

In order to develop more efficient HIV-resistant cells, fusion genes encoding the scFv formats of six new anti-HIV bNAbs (3BNC117, N6, PGT126, PGT128, 35O22, and 10E8) were constructed for cell surface expression through a GPI anchor. As shown in Figure 1(A), the scFv-encoding sequence was genetically fused with sequences encoding a His-tag and the GPI-anchoring signal sequence of decay accelerating factor (DAF) and then inserted into a self-inactivating lentiviral vector (pRRLsin.PPT.hPGK.WPRE). Herein, we refer to the constructs as GPI-3BNC117, GPI-N6, GPI-PGT126, GPI-PGT128, GPI-35O22, and GPI-10E8, respectively. The recombinant lentiviruses were generated by cotransfecting HEK293T cells with the transfer vector, a plasmid encoding vesicular stomatitis virus G glycoprotein (VSV-G), and a packaging plasmid encoding Gag/Pol/Rev. TZM-bl cells were then transduced with the recombinant lentiviruses and sorted by fluorescence-activated cell sorter (FACS) to obtain a cell population in which close to 100% of the cells expressed GPI-anchored scFvs. To confirm whether the transgenes were expressed on the cell surface through a GPI anchor, transduced TZM-bl cells were treated with phosphatidylinositol-specific phospholipase C (PI-PLC) and then stained with an anti-His tag antibody. The FACS analysis found that all of the six transgenes were efficiently expressed on the surface of transduced cells, but their expression obviously reduced after PI-PLC treatment (Figure 1(B)), verifying that each scFvs were tethered to the cell membrane via a GPI anchor.

It is important to know whether the cell surface anchoring and expression of GPI-scFvs interfere with the expression of HIV receptor and coreceptors. For this, transduced TZM-bl cells were stained with a PE-conjugated anti-human CD4, CCR5, or CXCR4 antibody and analyzed by FACS analysis. As shown in Figure 1(C), the fluorescent intensities of the TZM-bl cells transduced with GPI-scFvs were comparable to those of the parental TZM-bl cells (mock), indicating the expression of scFvs had no appreciable effect on the expression of the primary HIV-1 receptor CD4 and two coreceptors.

**Figure 2.**
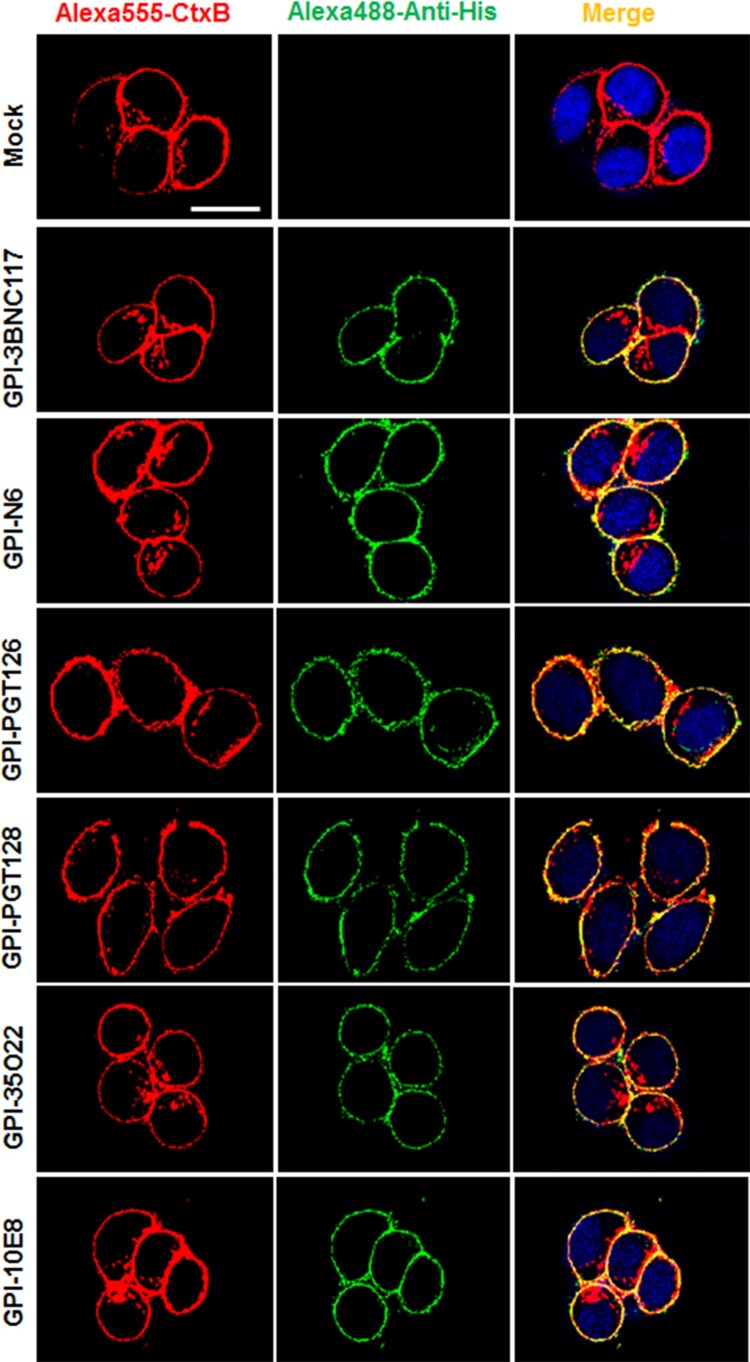
**Confocal analyses of GPI-scFvs in transduced TZM-bl cells.** Alexa555-CtxB stands for the lipid raft marker GM1 stained with Alexa 555-conjugated cholera toxin B subunit; Alexa488-Anti-His stands for the transduced cells stained with mouse anti-His tag antibody followed by Alexa 488-conjugated goat anti-mouse IgG antibody. Scale bar: 20 μm.

We previously showed that in the transduced cells GPI-anchored anti-gp120 nanobody m36.4 and fusion inhibitor peptide 2P23 were mainly located within the membrane lipid raft sites [26,27]. In this study, we also determined the distribution of GPI-scFvs by confocal microscopy. As shown in Figure 2, all GPI-scFvs were similarly colocalized with the lipid raft marker GM1 on the cell surface, confirming that they were indeed expressed in the lipid raft sites of the cell plasma membrane. Meanwhile, we did not observe any harmful effects of the constructs on the viability and growth of the transduced cells.

### GPI-scFvs confer various degrees of resistance to infections of divergent HIV-1 strains

We next determined the antiviral activity of new GPI-scFvs along with the previously-characterized control construct GPI-FluIgG03 that encodes scFv of an anti- influenza virus HA antibody. First, a panel of eight replication-competent HIV-1 strains with different tropisms was applied to infect TZM-bl cells transduced with the constructs. As shown in Table 1, GPI-FluIgG03-transduced TZM-bl cells were highly susceptible to infection with the HIV-1 strains including five CCR5-tropic viruses (JR-CSF, RHPA.c/2635, THRO.c/2626, CH077.t/2627, and MJ4), two CXCR4-tropic viruses (NL4-3 and LAI.2), and one dual-tropic virus (89.6). Interestingly, the TZM-bl cells expressing anti-HIV scFvs exhibited various degrees of resistance to HIV-1 infection: while GPI-10E8 rendered cells fully resistant to all the HIV-1 strains tested, other GPI-scFvs conferred resistance with much less capabilities. Specifically, the cells expressing GPI-PGT126 were permissive to 3 of 8 isolates with infectivity ranging from an average of 1%, 3%, and 90%, respectively; the cells expressing GPI-PGT128 were permissive to 6 of 8 isolates ranging from an average of 6-74% infectivity; the cells expressing GPI-3BNC117, GPI-N6, or GPI-35O22 were permissive to all of the 8 viruses ranging from an average of 30-102% infectivity.

**Table 1.** Inhibitory activities of GPI-scFvs against HIV-1 infection and Env-mediated cell-cell fusion.

HIV −1	Subtype	Tropism	Mean % infection ± SD						
			GPI-FluIgG03	GPI-3BNC117	*GPI-N6*	GPI-PGT126	GPI-PGT128	GPI-35O22	GPI-10E8
**Replication-competent virus**									
JR-CSF	B	CCR5	98 ± 2	44 ± 3	50 ± 6	0	0	75 ± 10	0
RHPA.c/2635	B	CCR5	97 ± 4	58 ± 7	57 ± 5	0	6 ± 1	81 ± 4	0
THRO.c/2626	B	CCR5	95 ± 5	87 ± 7	76 ± 1	90 ± 9	74 ± 13	87 ± 2	0
CH077.t/2627	B	CCR5	97 ± 5	50 ± 8	30 ± 1	3 ± 1	19 ± 1	69 ± 4	0
NL4-3	B	CXCR4	91 ± 3	102 ± 4	100 ± 2	0	14 ± 1	70 ± 4	0
LAI.2	B	CXCR4	92 ± 7	79 ± 8	77 ± 5	1	16 ± 1	55 ± 2	0
89.6	B	R5X4	104 ± 6	99 ± 3	54 ± 5	0	16 ± 1	87 ± 1	0
MJ4	C	CCR5	101 ± 4	79 ± 6	68 ± 4	0	0	71 ± 5	0
**"Global Panel" pseudovirus**									
398-F1_F6_20	A	CCR5	123 ± 4	128 ± 11	58 ± 6	0	0	91 ± 12	0
TRO.11	B	CCR5	104 ± 2	96 ± 11	53 ± 2	93 ± 7	84 ± 6	94 ± 3	0
X2278_C2_B6	B	CCR5	122 ± 5	82 ± 12	33 ± 3	49 ± 8	9	28 ± 2	0
CE1176_A3	C	CCR5	111 ± 4	101 ± 7	62 ± 8	0	0	87 ± 8	0
CE703010217_B6	C	CCR5	109 ± 4	94 ± 5	75 ± 1	4 ± 1	1	101 ± 1	0
HIV_25710-2.43	C	CCR5	113 ± 4	131 ± 18	49 ± 5	0	0	112 ± 8	0
X1632-S2-B10	G	CCR5	110 ± 2	104 ± 4	71 ± 9	103 ± 2	80 ± 6	104 ± 5	0
246_F3_C10_2	A/C	CCR5	110 ± 3	107 ± 14	38 ± 6	67 ± 10	2	85 ± 1	0
CNE8	A/E	CCR5	121 ± 4	127 ± 8	55 ± 4	57 ± 6	2	107 ± 9	0
CNE55	A/E	CCR5	119 ± 2	112 ± 6	59 ± 2	156 ± 3	139 ± 17	103 ± 11	1
CH119.10	B/C	CCR5	109 ± 3	117 ± 3	74 ± 1	50 ± 7	0	108 ± 6	0
BJOX002000.03	B/C	CCR5	108 ± 1	110 ± 12	59 ± 9	15 ± 3	0	104	0
VSV-G	NA	NA	100 ± 3	113 ± 7	103 ± 4	112 ± 5	89 ± 3	106 ± 8	97 ± 5
**Env -mediated cell fusion**									
398-F1_F6_20	A	CCR5	110 ± 5	58 ± 2	51 ± 4	2 ± 0	3 ± 1	60 ± 10	16 ± 3
92RW020	A	CCR5	109 ± 8	57 ± 4	53 ± 4	14 ± 1	19 ± 4	50 ± 9	6 ± 1
92UG037.8	A	CCR5	104 ± 3	54 ± 9	50 ± 8	1 ± 0	2 ± 0	48 ± 8	11 ± 1
NL4-3	B	CXCR4	112 ± 8	55 ± 5	47 ± 1	37 ± 3	58 ± 2	51 ± 11	9 ± 1
SF162	B	CCR5	102 ± 7	59 ± 2	47 ± 3	41 ± 1	36 ± 3	44 ± 7	3 ± 1
R3A	B	CCR5	107 ± 12	51 ± 7	55 ± 7	2 ± 0	16 ± 2	50 ± 11	17 ± 2
CNE11	B'	R5X4	110 ± 8	57 ± 5	51 ± 4	1 ± 0	2 ± 0	45 ± 9	21 ± 4
ZM53M.PB12	C	CCR5	101 ± 9	55 ± 6	50 ± 1	51 ± 9	65 ± 4	44 ± 11	12 ± 3
HIV_25710-2.43	C	CCR5	101 ± 6	53 ± 6	46 ± 2	0	0	52 ± 8	37 ± 4
GX11.13	A/E	CCR5	99 ± 7	50 ± 4	41 ± 0	13 ± 2	16 ± 1	50 ± 5	11 ± 1
CNE8	A/E	CCR5	103 ± 6	59 ± 7	52 ± 4	46 ± 6	51 ± 6	49 ± 11	40 ± 9
CNE55	A/E	CCR5	106 ± 4	51 ± 12	46 ± 5	57 ± 12	71 ± 7	47 ± 12	20 ± 3
CH70.1	B/C	R5X4	107 ± 6	80 ± 8	54 ± 8	42 ± 2	2 ± 0	57 ± 8	24 ± 7
CH119.10	B/C	CCR5	110 ± 7	79 ± 4	45 ± 1	35 ± 8	18 ± 4	50 ± 5	4 ± 1
BJOX002000.03	B/C	CCR5	111 ± 8	76 ± 9	67 ± 2	47 ± 7	42 ± 3	63 ± 8	28 ± 7

*The assay was performed in triplicate and repeated three times. Data are expressed as the means ±standard deviations (SD).

To testify the anti-HIV breadth and potency of GPI-scFvs in TZM-bl cells, a “global panel” of HIV-1 pseudoviruses, which included 12 divergent subtypes of viral Envs representing the world-wide AIDS epidemic, was further used [34]. As determined by a single-cycle infection assay, transduced cells also displayed various degrees of HIV-1 resistance, which were largely consistent with their activities in inhibiting the panel of replication-competent viruses (Table 1). As noted, GPI-10E8-modified cells completely blocked the infection of divergent HIV-1 subtypes, except for the pseudovirus CNE55, which infected the cells with an average infection rate of 1%. In regard to virus control, none of GPI-scFvs conferred resistance to vesicular stomatitis virus (VSV-G), indicating their antiviral specificity in transduced cells. In order to verify the GPI-mediated inhibitor anchoring, we also constructed a panel of lentiviral vectors that expressed secretory scFvs, and their antiviral activities were examined with the same panels of replication-competent HIV-1 and pseudoviruses. As shown, TZM-bl cells transduced with secretory scFvs could not effectively block HIV-1 infection (Table S1). As detected by Western blotting with an anti-His tag antibody, all the secretory scFvs presented only in the highly concentrated cell culture supernatants but not in the cell lysates (Fig. S1), which were consistent with the previous findings [19,26,35].

### GPI-scFvs confer various degrees of resistance to env-mediated cell–cell fusion and cell–cell HIV-1 transmission

**Figure 3.**
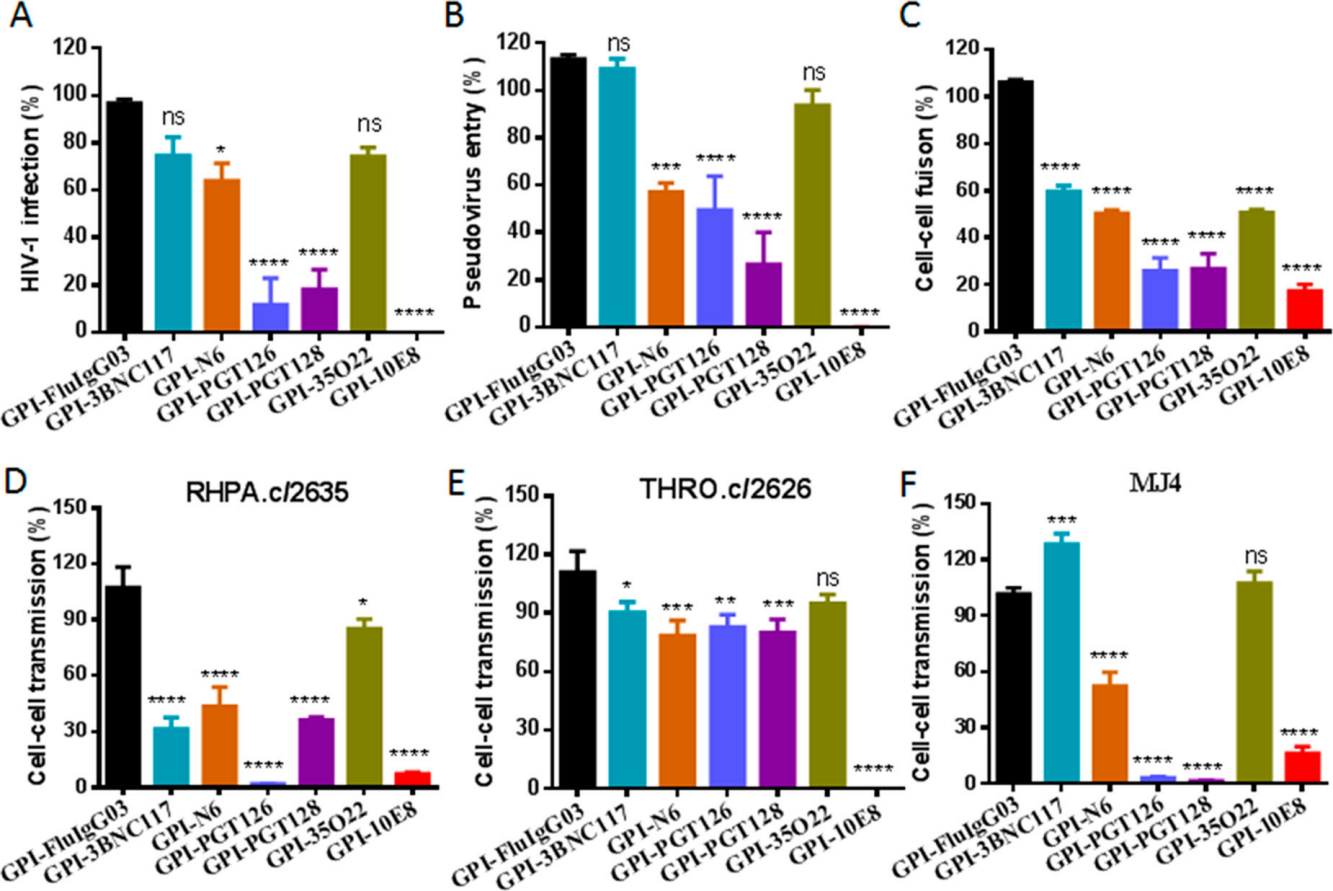
**Anti-HIV activities of GPI-scFvs in transduced cells.** The inhibitory activities of GPI-scFvs against a panel of replication-competent HIV-1 isolates **(A)**, the “global panel” of HIV-1 pseudoviruses **(B)**, and viral Env-mediated cell-cell fusion **(C)** in Table 1 were summarized for convenient overall view. Error bars indicate the means ± standard deviations (SD). **(D-F)** The inhibitory activities of GPI-scFvs on cell-cell HIV-1 transmission. TZM-bl cells expressing GPI-scFvs were used as a target and cocultured with CEMss-CCR5 donor cells that were preinfected with the HIV-1 isolates RHPA.c/2635 **(D)**, THRO.c/2626 (**E**) and MJ4 **(F)**. % cell-cell transmission was monitored by quantifying the production of the reporter luciferase in TZM-bl cells. Error bars indicate the means ± standard deviations (SD) from three independent experiments with triplicate samples, and statistical comparisons relative to the GPI-FluIgG03 control were conducted by ANOVA (ns, not significant; *, *P*< 0.05; **, *P* < 0.01; ***, *P* < 0.001; ****, *P* < 0.0001).

We next characterized the inhibitory capacity of GPI-scFvs on HIV-1 Env-mediated cell–cell fusion by a DSP-based cell fusion assay, in which HEK293T cells were transfected with a viral Env-expressing plasmid and a DSP_1-7_-expressing plasmid as effector cells, while 293FT cells stably expressing CCR5/CXCR4/DSP_8-11_ were transduced with GPI-scFvs as target cells (293FT_Target_). First, the cell surface expression of GPI-scFvs and their effects on the expression of CD4, CCR5, and CXCR4 were examined by FACS analysis. As shown in Fig. S2, all the transgenes were efficiently expressed on the surface of 293FT_Target_ and did not significantly affect the expression levels of the receptor and coreceptors. Similar to their inhibitory activities on replication-competent HIV-1 isolates and pseudoviruses, GPI-scFvs also exhibited different degrees of breadth and potency in blocking cell–cell fusion mediated by a panel of primary HIV-1 Envs with diverse subtypes and phenotypes (Table 1). Although GPI-10E8 showed an obviously attenuated efficiency relative to its inhibition on HIV-1 infection, it remained the most potent inhibitor against Env-mediated cell fusion. For an overall comparison, the data were also summarized in Figure 3(A-C).

We further determined the capacity of the membrane-anchored anti-HIV scFvs in blocking cell–cell HIV-1 transmission mediated by cell-associated virions with a protocol described previously. It is known that in the absence of a polycationic DEAE-dextran supplement, three cell-free HIV-1 strains, including two subtype B transmitted/founder viruses (RHPA.c/2635, THRO.c/2626) and one subtype C virus (MJ4), infect TZM-bl cells with a very low efficiency, whereas efficient cell–cell transmission of the cell-associated viruses (CEMss-CCR5^HIV+^) is independent of DEAE-dextran. Thus, TZM-bl cells were transduced with GPI-scFvs as target cells and then cocultured with HIV-infected donor cells (CEMss-CCR5^HIV+^ cells) without addition of DEAE-dextran. As shown in Figure 3(D-F), the TZM-bl cells expressing GPI-scFvs also displayed different resistance to the transmission of the three tested viruses from CEMss-CCR5^HIV+^ cells, and in comparison, GPI-10E8 was still a more effective inhibitor than other constructs in terms of the breadth and potency.

### GPI-10E8 renders human CD4^+^ T cells highly resistant to both CCR5- and CXCR4- HIV-1 strains and a selective survival advantage

**Figure 4.**
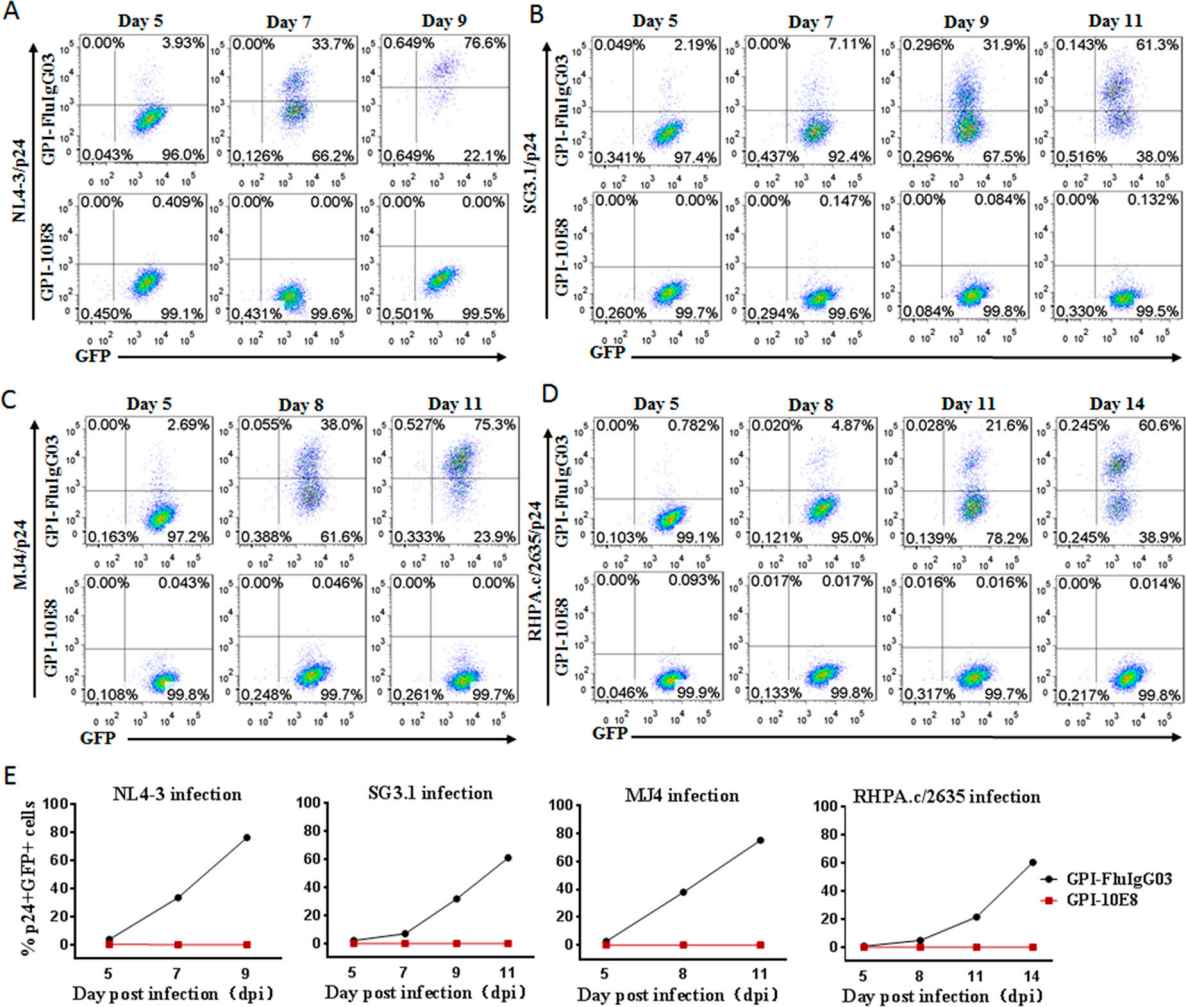
**Inhibitory activity of GPI-10E8 in transduced human CD4^+^ T cells against CXCR4-tropic and CCR5-tropic HIV-1 isolates.** CEMss-CCR5 cells transduced with GPI-10E8/GFP or GPI-FluIgG03/GFP were infected with 1,000 TCID_50_ of NL4-3 **(A)**, SG3.1 **(B)**, MJ4 **(C),** or RHPA.c/2635 **(D)** and intracellular HIV-1 p24 antigen and GFP expression were monitored over time by flow cytometry. **(E)** Infection curves of the transduced CEMss-CCR5 cells. Data from a representative experiment of at least two independent experiments are shown.

As shown above, GPI-10E8 could endow modified cells with potent resistance to diverse HIV-1 isolates. We next characterized whether GPI-10E8 could protect human CD4^+^ T cells from HIV-1 infection. To facilitate the monitoring of transduced cells, GPI-10E8 was genetically linked to green ﬂuorescent protein (GFP) through an internal 2A protein splicing signal in the lentiviral transfer vector pRRLsin-18.PPT.EF1α.WPRE (Fig. S3). The recombinant lentiviruses were packaged and transduced to CEMss-CCR5 cells. As detected by GFP and anti-His antibody, the GPI-10E8/GFP construct was efficiently expressed on the surface of CEMss-CCR5 cells and did not affect the expression of CD4, CCR5, and CXCR4 either. The transduced CEMss-CCR5 cells were first infected with two CXCR4-tropic HIV-1 strains (NL4-3 and SG3.1) and cultured in complete DMEM medium for 9 or 11 days, and the infected cells were monitored over time for intracellular HIV-1 p24 antigen and GFP expression by flow cytometry. As a control, GPI-FluIgG03/GFP-transduced CEMss-CCR5 cells showed 3.93%, 33.7%, and 76.6% p24- and GFP-double positive cells after NL4-3 challenge for 5, 7, and 9 days, respectively, and 2.19%, 7.11%, 31.9%, and 61.3% p24- and GFP-double positive cells after SG3.1 challenge for 5, 7, 9, 11 days, respectively (Figure 4(A,B), upper panels), indicating augmented infections over time; however, the CEMss-CCR5 cells expressing GPI-10E8/GFP had no or very minor (<0.409%) proportions of p24- and GFP-double positive cells during the observation (Figure 4(A,B), lower panels). The inhibitory activity of GPI-10E8/GFP was then assessed with two CCR5-tropic strains (MJ4 and RHPA.c/2635). Similarly, the GPI-FluIgG03/GFP-transduced CEMss-CCR5 cells were effectively infected by MJ4 or RHPA.c/2635 over time (Figure 4(C,D), upper panels); in sharp contrast, no or extremely few p24- and GFP-double positive cells were observed in the CEMss-CCR5 cells expressing GPI-10E8/GFP (Figure 4(C,D), lower panels) at 11 or 14 days. These results indicate that GPI-anchored 10E8 can render CD4^+^ T cells highly resistant to both CCR5- and CXCR4-tropic HIV-1 isolates. For clarity, the data are also shown in Figure 4(E).

**Figure 5.**
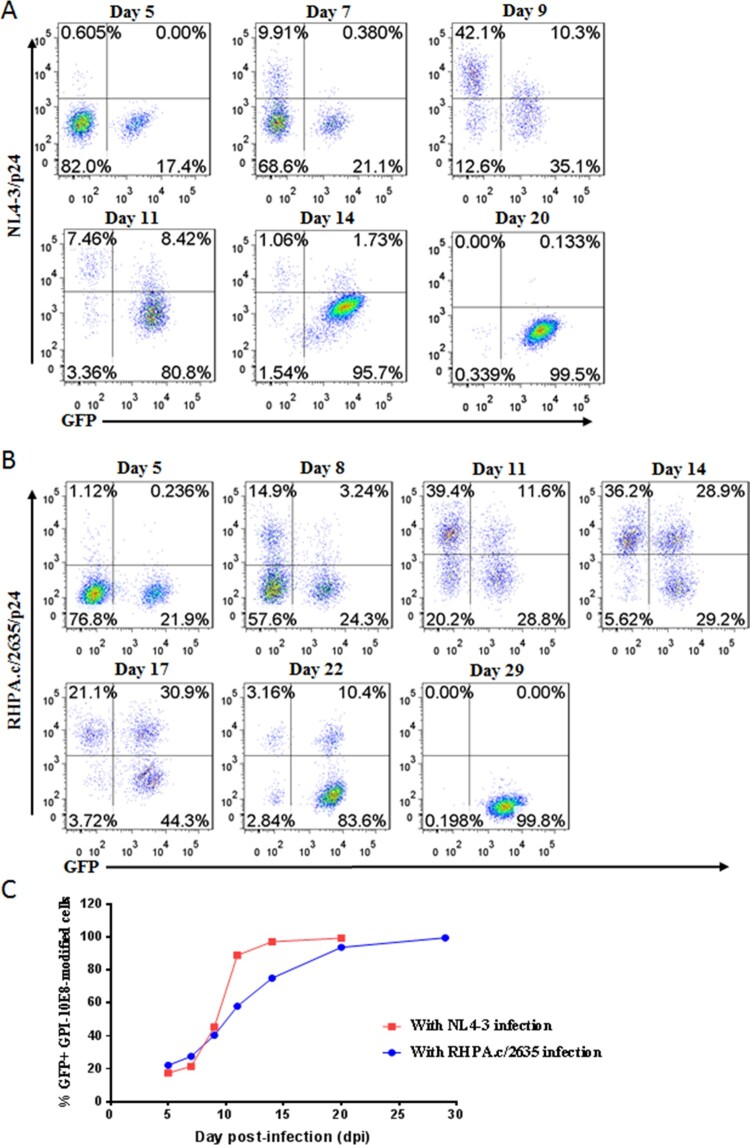
**Selective survival of human CD4^+^ T cells expressing GPI-10E8 during CXCR4-tropic or CCR5-tropic HIV-1 infection.** CEMss-CCR5 cells were transduced with GPI-10E8/GFP and mixed with untransduced cells at a ratio of approximately 20% GFP-positive cells. The mixed population was challenged with 1,000 TCID_50_ of CXCR4-tropic NL4-3 **(A)** or CCR5-tropic RHPA.c/2635 **(B)**, and the proportion of transgene-expressing cells was monitored over time by flow cytometry. **(C)** Survival curve of the transduced CEMss-CCR5 cells. Data from a representative experiment of at least two independent experiments are shown.

We further determined whether GPI-10E8-modified CEMss-CCR5 cells could be selectively enriched under HIV-1 infection. To this end, ∼20% GPI-10E8/GFP-transduced CEMss-CCR5 cells were mixed with untransduced cells and then subjected to HIV-1 challenge, followed by quantifying GFP expression with flow cytometry. As shown in Figure 5, the percentages of GFP-positive (GFP^+^) cells gradually increased over time in both CXCR4-tropic NL4-3 and CCR5-tropic RHPA.c/2635 infected cultures. Specifically, 99.5% of the cells expressed GPI-10E8/GFP after NL4-3 infection 20 days (Figure 5(A,C)), while 99.8% of the cells expressed GPI-10E8/GFP after RHPA.c/2635 infection 29 days (Figure 5(B,C)). Taken together, our results demonstrated that GPI-10E8 conferred strong selective survival and expansion advantages to human CD4^+^ T cells following HIV-1 infection.

### GPI-10E8 interferes with the HIV-1 Env processing and the infectivity of progeny viruses

Previous studies suggested that GPI-anchored anti-gp120 scFv X5 (GPI-X5) and nanobody (GPI-m36.4) can interfere with the expression and processing of viral Env glycoproteins and the infectivity of progeny HIV-1virions, underlying a multifaceted mode of antiviral action [26,36]. Herein, we also utilized similar experimental protocols to characterize the antiviral mechanism of GPI-10E8. First, a pcDNA3.1-based expression vector was constructed to exclude the potential impacts caused by the lentiviral vector. When the pcDNA3.1 construct was cotransfected with an HIV-1 provirus clone (NL4-3 or THRO.c/2626) into HEK293T cells, GPI-10E8 was finely expressed on the cell surface determined by FACS analysis (Fig. S4A); however, the gp120 amounts of both NL4-3 and THRO.c/2626 markedly decreased in the GPI-10E8-expressing cells, as detected by western blotting assay with a rabbit anti-gp120 polyclonal antibody (Fig. S4B). Comparing to the empty vector control and GPI-FluIgG03, the cotransfection of GPI-10E8 caused significant reduction in the ratio of gp120/gp160 (Fig. S4C). When probed with the human anti-gp41 monoclonal antibody 10E8, the gp41 amounts of the two HIV-1 strains also substantially decreased. Very interestingly, we repeatedly found that coexpression of NL4-3 and GPI-10E8 resulted in gp41 with a relatively smaller size in the Western blotting assay, which need to be characterized in detail.

We next analyzed the infectivity of progeny HIV-1 virions released from cells coexpressing GPI-10E8. To this end, cell culture supernatants containing equal amounts of the p24 antigen were applied to infect TZM-bl cells, and the relative virus infectivity was measured by quantifying luciferase activity in cell lysates. As shown in Fig. S4D, both NL4-3 and THRO.c/2626 released from cells expressing GPI-10E8 had dramatically decreased infectivity relative to the infection levels of the progeny viruses cotransfected with an empty vector or GPI-FluIgG03. Therefore, the results suggested that GPI-10E8 can impair HIV-1 Env processing and viral infectivity in transduced cells, consistent with the effect of GPI-m36.4 described previously.

### Design and validation of GPI-anchored bifunctional inhibitors targeting the gp41

**Figure 6.**
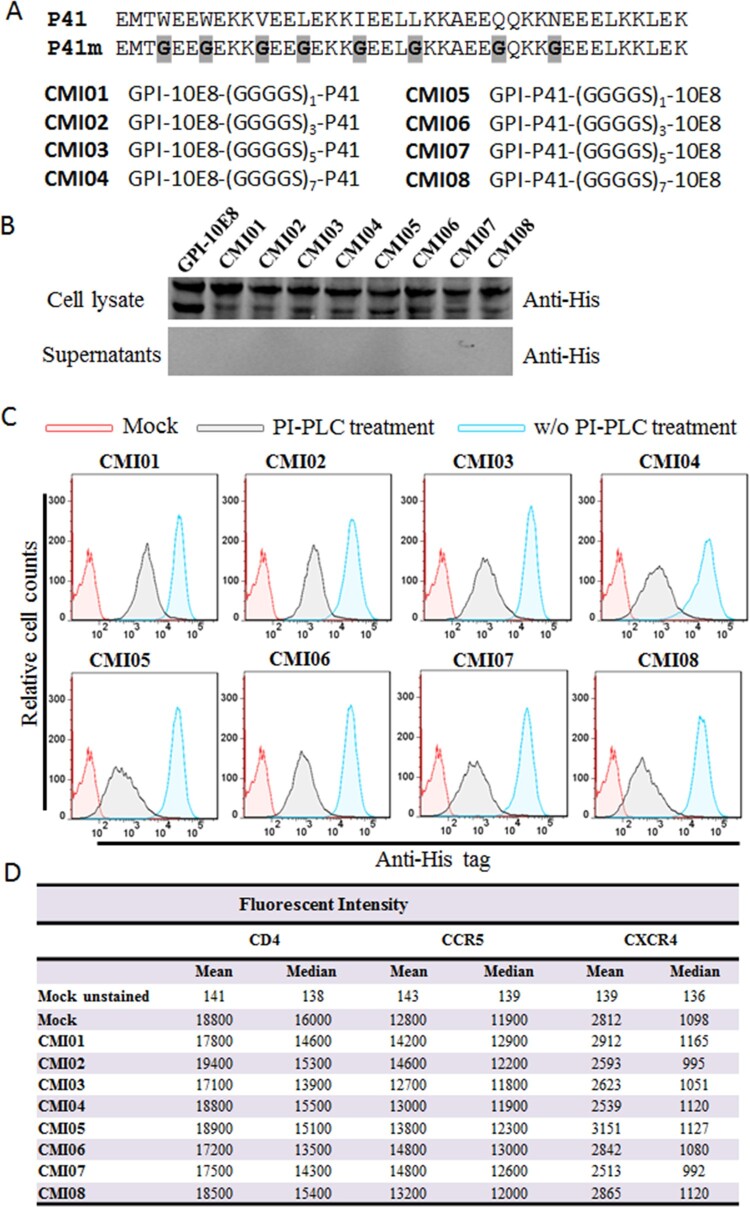
**Design and characterization of GPI-anchored bifunctional inhibitors targeting gp41. (A)** Design strategy. The sequence of a fusion inhibitor peptide P41 was genetically connected to the N- or C-termini of 10E8 scFv through a flexible (GGGGS)_n_ linker, generating eight GPI-anchored bifunctional constructs named CMI01 ∼ CMI08. **(B)** The expression of CMI constructs was detected in the cell lysates and culture supernatants of transduced TZM-bl cells by western blotting with an anti-His tag antibody. **(C)** Surface expression of CMI constructs in transduced TZM-bl cells with or without PI-PLC treatment was determined by FACS analysis using an anti-His tag antibody. **(D)** Effects of CMI constructs on the surface expression of CD4, CCR5, and CXCR4 in transduced TZM-bl cells were detected by FACS analysis using a PE-conjugated anti-human CD4, CCR5, or CXCR4 antibody and judged by fluorescence intensity.

Considering the high genetic variability and therapeutic evasion with HIV-1, we also devoted to develop GPI-anchored bifunctional inhibitors targeting the fusion protein gp41. In the design strategy, a fusion inhibitor peptide P41 was genetically connected to the N- or C-terminus of 10E8 scFv through a flexible (GGGGS)n linker (n=1, 3, 5, or 7), resulting in eight cell membrane-based inhibitors, named CMI01 ∼ CMI08 (Figure 6(A)). After the lentiviral vectors encoding the bifunctional inhibitors were constructed, the corresponding lentiviruses were similarly packaged and transduced into TZM-bl cells for characterization. As anticipated, the expression of the new constructs along with GPI-10E8 was readily detected by a western blotting assay in cell lysates but not in culture supernatants (Figure 6(B)); FACS analysis confirmed that each CMI inhibitor was efficiently expressed on the cell surface via a GPI anchor (Figure 6(C)) and did not significantly affect the expression of HIV receptor CD4 and coreceptors CCR5 and CXCR4 (Figure 6(D)). Similar to the results of GPI-scFvs above, the CMI constructs were also integrated into the plasma membrane lipid rafts via the DAF-derived GPI anchor, as judged by colocalization of Alexa488-Anti-His and Alexa555-CtxB with confocal microscopy (Fig. S5).

**Figure 7.**
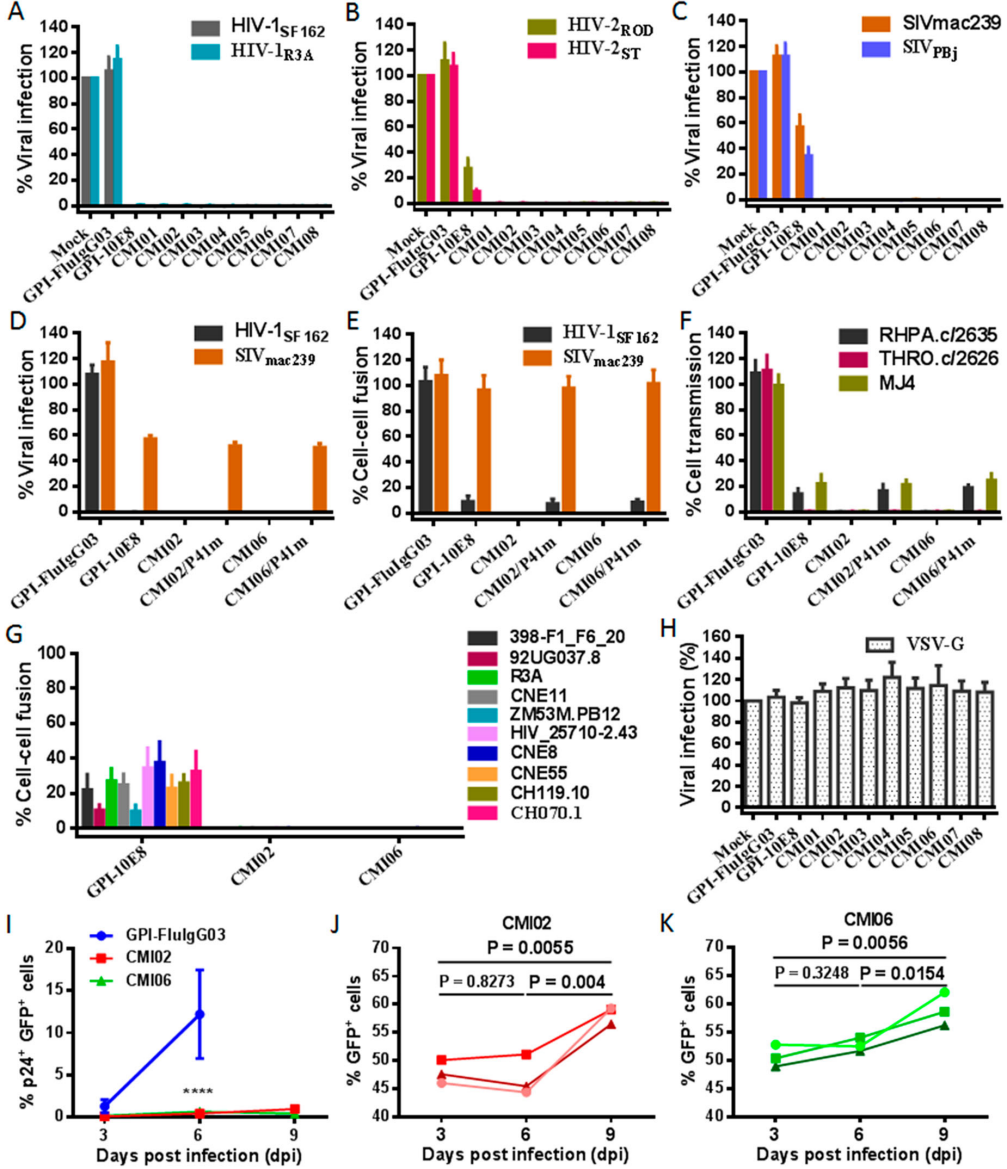
**Antiviral activity of GPI-anchored bifunctional inhibitors in transduced cells.** The inhibitory activities of CMI constructs against two HIV-1 pseudoviruses **(A)**, two replicative HIV-2 isolates **(B)**, and two SIV pseudoviruses **(C)**, as well as the inhibitory activities of the CMI02 and CMI06 mutants with the disruptive P41 mutations on the HIV-1_SF162_ and SIV_mac239_ pseudoviruses **(D)** and their Env-mediated cell-cell fusion **(E)**, and the cell-cell HIV-1 transmission of three replicative HIV-1 isolates **(F)** were respectively determined in transduced TZM-bl or 293FT_target_ cells. **(G)** The inhibitory activities of CMI02 and CMI06 constructs against the cell-cell fusion mediated by a panel of HIV-1 Envs that were partially resistant to GPI-10E8. **(H)** The inhibitory activities of GPI-10E8 and CMI constructs on VSV-G infection. **(I-K)** The anti-HIV activities of CMI02 and CMI06 in human primary CD4^+^ T cells. Transduced human primary CD4^+^ T cells were infected with 1,000 TCID_50_ of NL4-3, and intracellular p24 and GFP expression were monitored over time by flow cytometry. Infection curves of the transduced CD4^+^ T cells (**I**) represent the means ± SD from three independent experiments with triplicate samples, and statistical comparison was only performed between GPI-FluIgG03-ransduced cells and CMI02 or CMI06-transduced cells at day 6 after NL4-3 infection (****, *P* < 0.0001) as the most GPI-FluIgG03-transduced cells were dead at day 9. The survival curves of human CD4^+^ T cells transduced with CMI02 (**J**) and CMI06 (**K**) were statistically analyzed. The results of three independent experiments are shown with different colours of lines.

We were intrigued to know the anti-HIV activity of the CMI constructs in terms of their dual-functional destinations. As shown in Figure 7(A-C), transduction of each CMI construct rendered modified cells fully resistant to infections of two HIV-1 (R5-tropic SF162 and R5X4-tropic R3A), two HIV-2 (R5X4-tropic ROD and R5-tropic ST), and two SIV (R5-tropic mac239 and smmPBj) isolates. Because GPI-10E8 only partially blocked the HIV-2 and SIV infections, the results here suggested that the P41 peptide in the dual constructs critically contributed the potent antiviral activities. Interestingly, the connection order of two inhibitors and the linker length between them appeared to have no significant influence on the antiviral function of the CMI constructs. In order to confirm the role of the 10E8 part, the inhibitory activity of P41 in two representative constructs (CMI02 and CMI06) was abolished by mutating eight important amino acids in the peptide sequence (P41m in Figure 6(A)). As expected, two mutants, CMI02/P41m and CMI06/P41m, were well expressed on the surface of transduced TZM-bl and 293FT_Target_ cells without affecting the HIV receptor and coreceptors either (Fig. S6); however, they exhibited similar activities with GPI-10E8 in inhibiting the infections of HIV-1 SF162 and SIV mac239 (Figure 7(D)), indicating the disruption of P41 and functionality of 10E8 in the CMI constructs. The bifunctional effects of the constructs were further validated by comparing their inhibitions on the cell–cell fusion mediated by SF162 and mac239 Envs (Figure 7(E)) and the cell–cell transmission of three infectious HIV-1 isolates (RHPA.c/2635, THRO.c/2626, and MJ4) (Figure 7(F)). As shown, while CMI02 and CMI06 completely blocked the cell–cell fusion and transmission, CMI02/P41m and CMI06/P41m displayed comparable capacities with GPI-10E8. Combined, these results finely verified that the bifunctional CMI constructs possess superiority over GPI-10E8 in terms of anti-HIV potency and broad-spectrum. Consistently, CMI02 and CMI06 also rendered the modified 293FT_Target_ cells fully resistant to the fusion mediated by a panel of HIV-1 Envs that were resistant to GPI-10E8 (Figure 7(G)). As a virus control, none of the constructs inhibited VSV-G infection (Figure 7(H)). Moreover, we showed that 10E8-based constructs could efficiently prevent modified CEMss-CCR5 cells from fusion with HIV-1-infected donor cells, as indicated by syncytia counts in Fig. S7.

### CMI02 and CMI06 modified CEMss-CCR5 cells possess high resistance and survival advantage under HIV-1 infection

We next focused on evaluating the therapeutic efficacy of the bifunctional inhibitors in human CD4^+^ T cells. CMI02 and CMI06 were accordingly constructed with the transfer vector pRRLsin-18.PPT.EF1α.WPRE to efficiently express CMI02/GFP or CMI06/GFP in transduced CEMss-CCR5 cells, which did not affect the surface expression of CD4, CCR5, and CXCR4 either (Fig. S8). Similarly, the transduced cells were inoculated with three CXCR4-tropic (NL4-3, SG3.1, and LAI.2) and three CCR5-tropic (RHPA.c/2635, TRJO.c/2851, and MJ4) viruses, and infections were monitored over time by detecting intracellular HIV-1 p24 antigen and GFP expression. While CEMss-CCR5 cells expressing GPI-FluIgG03/GFP exhibited increased infections over time, no or very minor p24+ and GFP+ cells were observed among the cells transduced with CMI02/GFP or CMI06/GFP (Fig. S9-S10).

Subsequently, we examined the selective survival ability of CEMss-CCR5 cells expressing a dual inhibitor. Thus, a mixture containing ∼20% transduced cells was generated and then challenged by NL4-3 or MJ4. As monitored by GFP expression, the proportion of transduced cells dramatically increased over time. Specifically, CMI02 and CMI06-transduced cells had increased to 99.7% and 99.524%, respectively, after NL4-3 infection 21 days and increased to 98.956% and 99.048%, respectively, after MJ4 infection 20 days (Fig. S11). For clarity, the infection and survival curves of the transduced CEMss-CCR5 are also summarized and shown in Fig. S12.

### CMI02 and CMI06 are highly effective in transduced human primary CD4^+^ T cells

We further evaluated CMI02 and CMI06 for their functionalities in human primary CD4^+^ T cells. To this end, purified CD4^+^ T cells were stimulated with anti-CD3/CD28-coated beads and then transduced with the CMI02 and CMI06 vectors. As shown in Fig. S13(A,B), both CMI02 and CMI06 could be efficiently expressed in transduced CD4^+^ T cells without appreciable cytotoxicity. To examine the inhibitory activity of CMI02 and CMI06, transduced cells were inoculated with HIV-1 NL4-3 and intracellular p24 antigen along with GFP expression were monitored every 3 days by FACS analysis. As indicated by p24 expression, while 20.1% of untransduced cells and 13.96% of GPI-FluIgG03/GFP-transduced cells were infected by NL4-3 at day 6 post-challenge and then the cells gradually died, only ∼4-5% of CMI02- or CMI06-transduced cells in a total population were infected after NL4-3 challenge 6 or 9 days (Fig. S13C). It was verified that of cells transduced with CMI02 and CMI06 <1% were p24- and GFP-double positive (p24^+^GFP^+^) after NL4-3 infection 6 or 9 days (Figure 7I) and that the percentage of transduced-cells (GFP^+^) significantly increased over time (Figure 7(J,K)). Taken together, the results suggested that CMI02 and CMI06 can efficiently modify human primary CD4^+^ T cells with high resistance to HIV-1 infection and survival advantage.

## Discussion

In this study, we continued our efforts to develop a more efficient strategy for HIV-1 gene therapy and achieved significant findings. First, a panel of newly generated HIV-1 neutralizing antibodies was comprehensively characterized as membrane-bound scFv inhibitors and found that GPI-anchored 10E8 was the most effective in the inhibitions of cell-free HIV-1 infection, cell-associated HIV-1 transmission, and viral Env-mediated cell–cell fusion. Second, we showed that GPI-10E8 also interfered with the processing of viral Env in transduced cells and attenuated the infectivity of progeny viruses, verifying a multifaceted mechanism of action similar to that of GPI-anchored domain antibody m36.4 characterized previously. Third, a group of bifunctional inhibitors, designated CMI01∼CMI08, were created as cell membrane-based inhibitors by linking 10E8 scFv and a 41-mer fusion inhibitor peptide P41, which rendered modified cells fully resistant to infections of HIV-1, HIV-2, and simian immunodeficiency virus (SIV). Fourth, GPI-10E8 and its bifunctional derivatives (CMI02 and CMI06) were highly effective in transduced human CEMss-CCR5 cells to inhibit both CCR5- and CXCR4-tropic HIV-1 isolates and the modified cells experienced a robust survival selection under HIV-1 infection. Fifth, we verified the potent anti-HIV activities of CMI02 and CMI06 in transduced human primary CD4^+^ T cells without cytotoxicity. Therefore, we conclude that our current studies characterize 10E8 as a novel antibody for generating HIV-resistant cells and its bifunctional derivatives with a fusion-inhibitory peptide possess more potent and broad-spectrum antiviral activity, thereby providing new strategies for HIV gene therapy that can be used alone or in combination.

In the last two decades, a variety of strategies, including RNA-based and protein-based approaches or their combinations, have been explored for cure or functional cure of HIV-1 infection, and of them a group of approaches is currently being investigated in clinical settings [18,37–40]. Promisingly, gene therapy establishing a subset of HIV-resistant target cells has shown therapeutic efficacy and has high potential to mimic the cases known as the “Berlin patient” and the “London patient”. Given the disappointed results of allogeneic transplantation of CCR5 gene-edited HSCs in patients and concerns over the deleterious effects of the CCR5Δ32 mutation, we generally consider that knocking in antiviral genes is a safer and more efficient way than the deletion or disruption of host cell genes to modify cells for resistance, and especially, inhibitors that block viral entry step possess prominent advantages. Because GPI anchoring in nature is a typical posttranslational modification process and many GPI-linked proteins are directed to lipid rafts where host the HIV receptors and serve as gateways for HIV entry and budding, we prefer anchoring an HIV entry inhibitor such as antibody or peptide to the cell membrane through a GPI anchor as a technical platform. For this, our previous studies verified that GPI-anchoring signal sequence of decay accelerating factor (DAF) is viable to position antibodies and peptides to the cell membrane lipid raft sites [26,27].

Broadly neutralizing antibodies (bNAbs) have been extensively explored in HIV vaccine development and as soluble antibody drugs [23,25,41–44]. In an advanced stage, several bNAbs with potent and broad-spectrum anti-HIV activity are currently being investigated in clinical trials; however, it should be realized that very few studies have focused on developing antibody-based resistant cells for HIV gene therapy. By using a similar GPI-anchoring platform, Zhou and coworkers previously characterized a group of human and llama neutralizing antibodies or functional fragments targeting different gp120 or gp41 epitopes, including b12 and JM2 (gp120 CD4bs epitope), X5, 48d, E51, and JM4 (gp120 CD4i epitope), PG9 and PG16 (gp120 V2/V3-glycan epitope), 4E10 (gp41 MPER epitope) and TG15 and AB31 (gp41 cluster II or III epitopes) [19–21]. In comparison, GPI-scFvs targeting the CD4i epitope (GPI-X5) and MPER epitope (GPI-4E10) possessed higher inhibitory potency and breadth. It was found that GPI-anchored variable region (VHH) of CD4i-specific JM4 but not that of CD4bs-specific JM2 was able to render target cells resistant to both cell-free and cell-associated HIV-1 isolates, although two heavy chain-only llama antibodies displayed comparable anti-HIV activities in a soluble form [21], revealing that the epitope specificity critically determines the functionality of GPI-anchored anti-HIV antibodies in distinct formats. For a potential application in HIV-1 gene therapy, GPI-X5 was further characterized for its therapeutic efficacy in a humanized mouse infection model as well as the underlying mechanism of action [22,36]. In our previous studies, we also demonstrated that the CD4i epitope-specific domain antibody m36.4 when used as a GPI-anchored inhibitor was highly effective against both cell-free and cell-associated HIV-1 isolates [26]. Herein, our studies verified that the MPER epitope-specific 10E8 also rendered modified cells with the most potent and broad-spectrum anti-HIV efficacy, whereas GPI-anchored bNAbs targeting the CD4bs epitope (3BNC117 and N6), the V3-glycan epitope (PGT126 and PGT128), and the gp120/gp41 interface epitope (35O22) showed limited effectiveness. Taken these data together, we could realize that two classes of antibodies targeting the CD4i epitope on gp120 subunit and MPER epitope on gp41 subunit would confer the most broad and potent inhibitory activity when being genetically anchored to the cell membrane through a GPI attachment signal, thus being ideal membrane-based entry inhibitors for generating HIV-resistant cells. At the same time, we also realize that some scFvs are not suitable as GPI-anchored inhibitors due to their markedly reduced neutralizing activities compared to the parental IgG antibodies, as recently reported by Dorsten et al [45].

As well-known, the current generation bNAbs possess greatly increased anti-HIV activities relative to the first generation HIV-neutralizing antibodies. Such is indeed the case for 10E8, which possesses much better neutralizing potency and breadth than 4E10 [33], although they target the overlapping MPER epitopes, and thus, it is conceivable that GPI-10E8 is a more efficient strategy for generating HIV-resistant cells that can be used alone or in combination with other genetic intervention constructs. Nonetheless, we observed that the inhibitory activities of GPI-10E8 against HIV-1 Env-mediated cell–cell fusion and cell–cell virus transmission were visibly decreased compared to its potency in inhibiting divergent cell-free viruses. Consistently, the previous studies also showed that MPER-directed bNAbs had a relatively lower capacity to inhibit HIV-1 cell–cell spread and were strain- and epitope-dependent [46–48]. Moreover, it was found that MPER adopts distinct structural/functional forms during cell-free and cell-to-cell viral spread, with the latter form being unavailable for interaction with bNAbs [49]. In sharp contrast, the membrane-bound fusion inhibitor peptides such as 2P23 and C34, which exclusively target the upstream N-terminal heptad repeat (NHR) site of gp41, have been validated to be highly effective against both cell-free and cell-associated HIV-1/2 isolates [16,17,27]. Considering the weakness of GPI-10E8 and high variability of HIV-1, it is of significance to develop a bi- or multi-functional GPI-anchored inhibitor, which can improve the anti-HIV efficacy and genetic barrier to inducing resistance to inhibitor *pe se*. In this regard, the previous studies reported that escape mutant viruses readily emerged during the monotherapy with single bNAb [41,42,44,50]; however, mutations in gp41 that confer one inhibitor resistance may enhance sensitivity to another gp41 inhibitor. For example, it was found that 10E8 exhibited increased activity in neutralizing HIV-1 mutants resistant to a fusion inhibitor peptide (C34) [51]. In the present study, the successful design of the CMI constructs by genetically linking 10E8 scFv and fusion-inhibitory peptide P41 did offer GPI-anchored bifunctional inhibitors with multiple advantages, including its inhibitory potency and breadth as well as potential genetic resistance barrier. The bifunctional constructs also provide new arsenals fighting HIV-2, which has already caused increased infections worldwide [52]. In addition to their high antiviral efficacy, we are still intrigued to understand the mechanism of action of the CMI constructs. Especially, how two tandem parts exert their virus-inhibitions irrespective of the connection direction and linker size remains to be elucidated; in other words, the structure–function relationship (SAR) of the dual inhibitors would benefit to explore the mechanism of HIV infection and thus need our future studies.

In one-sentence conclusion, our studies identified 10E8 as a powerful GPI-anchored antibody for generating HIV-resistant cells and for the first time we designed GPI-anchored bifunctional HIV entry inhibitors targeting the conservative gp41 fusion protein. Definitely, the *in vivo* therapeutic efficacy and potential side-effects of the new constructs as a genetic intervention should be carefully characterized before they can be evaluated in clinical settings.

## Supplementary Material

Supplemental Material

## Data Availability

All data are fully available without restriction.

## References

[1] Barre-Sinoussi F, Ross AL, Delfraissy JF. Past, present and future: 30 years of HIV research. Nat Rev Microbiol. 2013;11:877–883. 24162027 10.1038/nrmicro3132

[2] Collier DA, Monit C, Gupta RK. The impact of HIV-1 drug escape on the global treatment landscape. Cell Host Microbe. 2019;26:48–60. 31295424 10.1016/j.chom.2019.06.010

[3] Hutter G, Nowak D, Mossner M, et al. Long-term control of HIV by CCR5 Delta32/Delta32 stem-cell transplantation. N Engl J Med. 2009;360:692–698. 19213682 10.1056/NEJMoa0802905

[4] Gupta RK, Abdul-Jawad S, McCoy LE, et al. HIV-1 remission following CCR5Delta32/Delta32 haematopoietic stem-cell transplantation. Nature. 2019;568:244–248. 30836379 10.1038/s41586-019-1027-4 PMC7275870

[5] Peterson CW, Kiem HP. Lessons from London and Berlin: designing A scalable gene therapy approach for HIV cure. Cell Stem Cell. 2019;24:685–687. 31051132 10.1016/j.stem.2019.04.010

[6] Henrich TJ, Hanhauser E, Marty FM, et al. Antiretroviral-free HIV-1 remission and viral rebound after allogeneic stem cell transplantation: report of 2 cases. Ann Intern Med. 2014;161:319–327. 25047577 10.7326/M14-1027 PMC4236912

[7] Cummins NW, Rizza S, Litzow MR, et al. Extensive virologic and immunologic characterization in an HIV-infected individual following allogeneic stem cell transplant and analytic cessation of antiretroviral therapy: A case study. PLoS Med. 2017;14:e1002461. 29182633 10.1371/journal.pmed.1002461 PMC5705162

[8] Xu L, Wang J, Liu Y, et al. CRISPR-Edited Stem cells in a patient with HIV and acute lymphocytic leukemia. N Engl J Med. 2019;381:1240–1247. 31509667 10.1056/NEJMoa1817426

[9] Kordelas L, Verheyen J, Beelen DW, et al. Shift of HIV tropism in stem-cell transplantation with CCR5 Delta32 mutation. N Engl J Med. 2014;371:880–882. 10.1056/NEJMc1405805 25162903

[10] Glass WG, McDermott DH, Lim JK, et al. CCR5 deficiency increases risk of symptomatic West Nile virus infection. J Exp Med. 2006;203:35–40. 16418398 10.1084/jem.20051970 PMC2118086

[11] Kindberg E, Mickiene A, Ax C, et al. A deletion in the chemokine receptor 5 (CCR5) gene is associated with tickborne encephalitis. J Infect Dis. 2008;197:266–269. 18179389 10.1086/524709

[12] Hayashi T, MacDonald LA, Takimoto T. Influenza A virus protein PA-X contributes to viral growth and suppression of the Host antiviral and immune responses. J Virol. 2015;89:6442–6452. 25855745 10.1128/JVI.00319-15 PMC4474289

[13] Engelman A, Cherepanov P. The structural biology of HIV-1: mechanistic and therapeutic insights. Nat Rev Microbiol. 2012;10:279–290. 22421880 10.1038/nrmicro2747 PMC3588166

[14] Hildinger M, Dittmar MT, Schult-Dietrich P, et al. Membrane-anchored peptide inhibits human immunodeficiency virus entry. J Virol. 2001;75:3038–3042. 11222732 10.1128/JVI.75.6.3038-3042.2001 PMC115933

[15] Egelhofer M, Brandenburg G, Martinius H, et al. Inhibition of human immunodeficiency virus type 1 entry in cells expressing gp41-derived peptides. J Virol. 2004;78:568–575. 14694088 10.1128/JVI.78.2.568-575.2004 PMC368739

[16] Liu L, Wen M, Zhu Q, et al. Glycosyl phosphatidylinositol-anchored C34 peptide derived from human immunodeficiency virus type 1 Gp41 Is a potent entry inhibitor. J Neuroimmune Pharmacol. 2016;11:601–610. 27155865 10.1007/s11481-016-9681-x PMC5577817

[17] Leslie GJ, Wang J, Richardson MW, et al. Potent and broad Inhibition of HIV-1 by a peptide from the gp41 heptad repeat-2 domain conjugated to the CXCR4 amino terminus. PLoS Pathog. 2016;12:e1005983. 27855210 10.1371/journal.ppat.1005983 PMC5113989

[18] Falkenhagen A, Joshi S. Genetic strategies for HIV treatment and prevention. Mol Ther Nucleic Acids. 2018;13:514–533. 30388625 10.1016/j.omtn.2018.09.018 PMC6205348

[19] Wen M, Arora R, Wang H, et al. GPI-anchored single chain Fv--an effective way to capture transiently-exposed neutralization epitopes on HIV-1 envelope spike. Retrovirology. 2010;7:79. 20923574 10.1186/1742-4690-7-79 PMC2959034

[20] Liu L, Wen M, Wang W, et al. Potent and broad anti-HIV-1 activity exhibited by a glycosyl-phosphatidylinositol-anchored peptide derived from the CDR H3 of broadly neutralizing antibody PG16. J Virol. 2011;85:8467–8476. 21715497 10.1128/JVI.00520-11 PMC3165811

[21] Liu L, Wang W, Matz J, et al. The Glycosylphosphatidylinositol-Anchored Variable region of llama heavy chain-only antibody JM4 efficiently blocks both cell-free and T cell-T cell transmission of human Immunodeficiency Virus Type 1. J Virol. 2016;90:10642–10659. 27654286 10.1128/JVI.01559-16 PMC5110191

[22] Ye C, Wang W, Cheng L, et al. Glycosylphosphatidylinositol-Anchored Anti-HIV scFv efficiently Protects CD4 T cells from HIV-1 infection and deletion in hu-PBL mice. J Virol. 2017;91:e01389–16. 27881659 10.1128/JVI.01389-16 PMC5244347

[23] Stephenson KE, Wagh K, Korber B, et al. Vaccines and broadly neutralizing antibodies for HIV-1 prevention. Annu Rev Immunol. 2020;38:673–703. 32340576 10.1146/annurev-immunol-080219-023629 PMC7375352

[24] Barouch DH. A step forward for HIV vaccines. Lancet HIV. 2018;5:e338–e339. 29898871 10.1016/S2352-3018(18)30095-X PMC6093284

[25] Alter G, Barouch D. Immune correlate-guided HIV vaccine design. Cell Host Microbe. 2018;24:25–33. 30001521 10.1016/j.chom.2018.06.012 PMC6114134

[26] Jin H, Tang X, Li L, et al. Generation of HIV-resistant cells with a single-domain antibody: implications for HIV-1 gene therapy. Cell Mol Immunol. 2021;18:660–674. 33462383 10.1038/s41423-020-00627-y PMC7812570

[27] Tang X, Jin H, Chen Y, et al. A membrane-anchored short-peptide fusion inhibitor fully Protects target cells from infections of HIV-1, HIV-2, and simian immunodeficiency virus. J Virol. 2019;93:e01177–19. 10.1128/JVI.01177-19 PMC6819927 31462566

[28] Scheid JF, Mouquet H, Ueberheide B, et al. Sequence and structural convergence of broad and potent HIV antibodies that mimic CD4 binding. Science. 2011;333:1633–1637. 21764753 10.1126/science.1207227 PMC3351836

[29] Huang J, Kang BH, Ishida E, et al. Identification of a CD4-binding-site antibody to HIV that evolved near-Pan neutralization breadth. Immunity. 2016;45:1108–1121. 27851912 10.1016/j.immuni.2016.10.027 PMC5770152

[30] Pejchal R, Doores KJ, Walker LM, et al. A potent and broad neutralizing antibody recognizes and penetrates the HIV glycan shield. Science. 2011;334:1097–1103. 21998254 10.1126/science.1213256 PMC3280215

[31] Walker LM, Huber M, Doores KJ, et al. Broad neutralization coverage of HIV by multiple highly potent antibodies. Nature. 2011;477:466–470. 21849977 10.1038/nature10373 PMC3393110

[32] Huang J, Kang BH, Pancera M, et al. Broad and potent HIV-1 neutralization by a human antibody that binds the gp41-gp120 interface. Nature. 2014;515:138–142. 25186731 10.1038/nature13601 PMC4224615

[33] Huang J, Ofek G, Laub L, et al. Broad and potent neutralization of HIV-1 by a gp41-specific human antibody. Nature. 2012;491:406–412. 23151583 10.1038/nature11544 PMC4854285

[34] deCamp A, Hraber P, Bailer RT, et al. Global panel of HIV-1 Env reference strains for standardized assessments of vaccine-elicited neutralizing antibodies. J Virol. 2014;88:2489–2507. 24352443 10.1128/JVI.02853-13 PMC3958090

[35] Liu L, Wang W, Yang L, et al. Trimeric glycosylphosphatidylinositol-anchored HCDR3 of broadly neutralizing antibody PG16 is a potent HIV-1 entry inhibitor. J Virol. 2013;87:1899–1905. 23152526 10.1128/JVI.01038-12 PMC3554188

[36] Misra A, Gleeson E, Wang W, et al. Glycosyl-Phosphatidylinositol-Anchored anti-HIV Env Single-Chain Variable Fragments interfere with HIV-1 Env processing and viral infectivity. J Virol. 2018;92:e02080–17. 29321330 10.1128/JVI.02080-17 PMC5972903

[37] de Mendoza C, Barreiro P, Benitez L, et al. Gene therapy for HIV infection. Expert Opin Biol Ther. 2015;15:319–327. 25323559 10.1517/14712598.2015.967208

[38] Burke BP, Levin BR, Zhang J, et al. Engineering cellular resistance to HIV-1 infection In vivo using a dual therapeutic lentiviral vector. Mol Ther Nucleic Acids. 2015;4:e236. 25872029 10.1038/mtna.2015.10

[39] Herrera-Carrillo E, Berkhout B. Potential mechanisms for cell-based gene therapy to treat HIV/AIDS. Expert Opin Ther Targets. 2015;19:245–263. 25388088 10.1517/14728222.2014.980236

[40] Herrera-Carrillo E, Berkhout B. Bone marrow gene therapy for HIV/AIDS. Viruses. 2015;7:3910–3936. 26193303 10.3390/v7072804 PMC4517133

[41] Caskey M, Klein F, Lorenzi JC, et al. Viraemia suppressed in HIV-1-infected humans by broadly neutralizing antibody 3BNC117. Nature. 2015;522:487–491. 25855300 10.1038/nature14411 PMC4890714

[42] Lynch RM, Boritz E, Coates EE, et al. Virologic effects of broadly neutralizing antibody VRC01 administration during chronic HIV-1 infection. Sci Transl Med. 2015;7:319ra206. 10.1126/scitranslmed.aad5752 PMC12366723 26702094

[43] Bar KJ, Sneller MC, Harrison LJ, et al. Effect of HIV antibody VRC01 on viral rebound after treatment interruption. N Engl J Med. 2016;375:2037–2050. 27959728 10.1056/NEJMoa1608243 PMC5292134

[44] Caskey M, Schoofs T, Gruell H, et al. Antibody 10-1074 suppresses viremia in HIV-1-infected individuals. Nat Med. 2017;23:185–191. 28092665 10.1038/nm.4268 PMC5467219

[45] van Dorsten RT, Lambson BE, Wibmer CK, et al. Neutralization breadth and potency of Single-Chain variable fragments derived from broadly neutralizing antibodies targeting multiple epitopes on the HIV-1 envelope. J Virol. 2020;94:e01533-19. 31619559 10.1128/JVI.01533-19 PMC6955269

[46] Reh L, Magnus C, Schanz M, et al. Capacity of broadly neutralizing antibodies to inhibit HIV-1 cell-cell transmission Is strain- and epitope-dependent. PLoS Pathog. 2015;11:e1004966. 26158270 10.1371/journal.ppat.1004966 PMC4497647

[47] Gombos RB, Kolodkin-Gal D, Eslamizar L, et al. Inhibitory effect of individual or combinations of broadly neutralizing antibodies and antiviral reagents against cell-free and cell-to-cell HIV-1 transmission. J Virol. 2015;89:7813–7828. 25995259 10.1128/JVI.00783-15 PMC4505680

[48] Malbec M, Porrot F, Rua R, et al. Broadly neutralizing antibodies that inhibit HIV-1 cell to cell transmission. J Exp Med. 2013;210:2813–2821. 24277152 10.1084/jem.20131244 PMC3865481

[49] Narasimhulu VGS, Bellamy-McIntyre AK, Laumaea AE, et al. Distinct functions for the membrane-proximal ectodomain region (MPER) of HIV-1 gp41 in cell-free and cell-cell viral transmission and cell-cell fusion. J Biol Chem. 2018;293:6099–6120. 29496992 10.1074/jbc.RA117.000537 PMC5912456

[50] Scheid JF, Horwitz JA, Bar-On Y, et al. HIV-1 antibody 3BNC117 suppresses viral rebound in humans during treatment interruption. Nature. 2016;535:556–560. 27338952 10.1038/nature18929 PMC5034582

[51] Alam M, Kuwata T, Shimura K, et al. Enhanced antibody-mediated neutralization of HIV-1 variants that are resistant to fusion inhibitors. Retrovirology. 2016;13:70. 27670680 10.1186/s12977-016-0304-7 PMC5037607

[52] Visseaux B, Damond F, Matheron S, et al. Hiv-2 molecular epidemiology. Infect Genet Evol. 2016;46:233–240. 27530215 10.1016/j.meegid.2016.08.010

