# Supplementary figures and images for: Cell membrane-anchored anti-HIV single-chain antibodies and bifunctional inhibitors targeting the gp41 fusion protein: new strategies for HIV gene therapy
Source: Emerg Microbes Infect. 2021 Dec 21;11(1):30–49. doi: 10.1080/22221751.2021.2011616 (PMC8735881; doi:10.1080/22221751.2021.2011616)

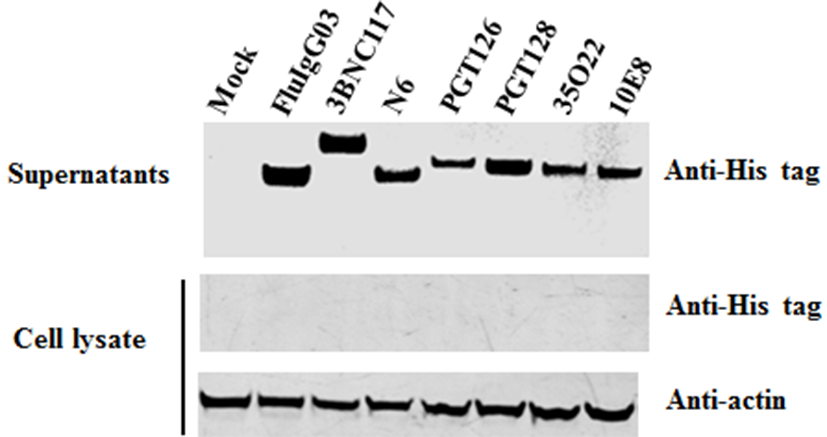

Supplement: Supplemental Material [file TEMI_A_2011616_SM4340.zip › Supplementary files/Fig. S1.tif]

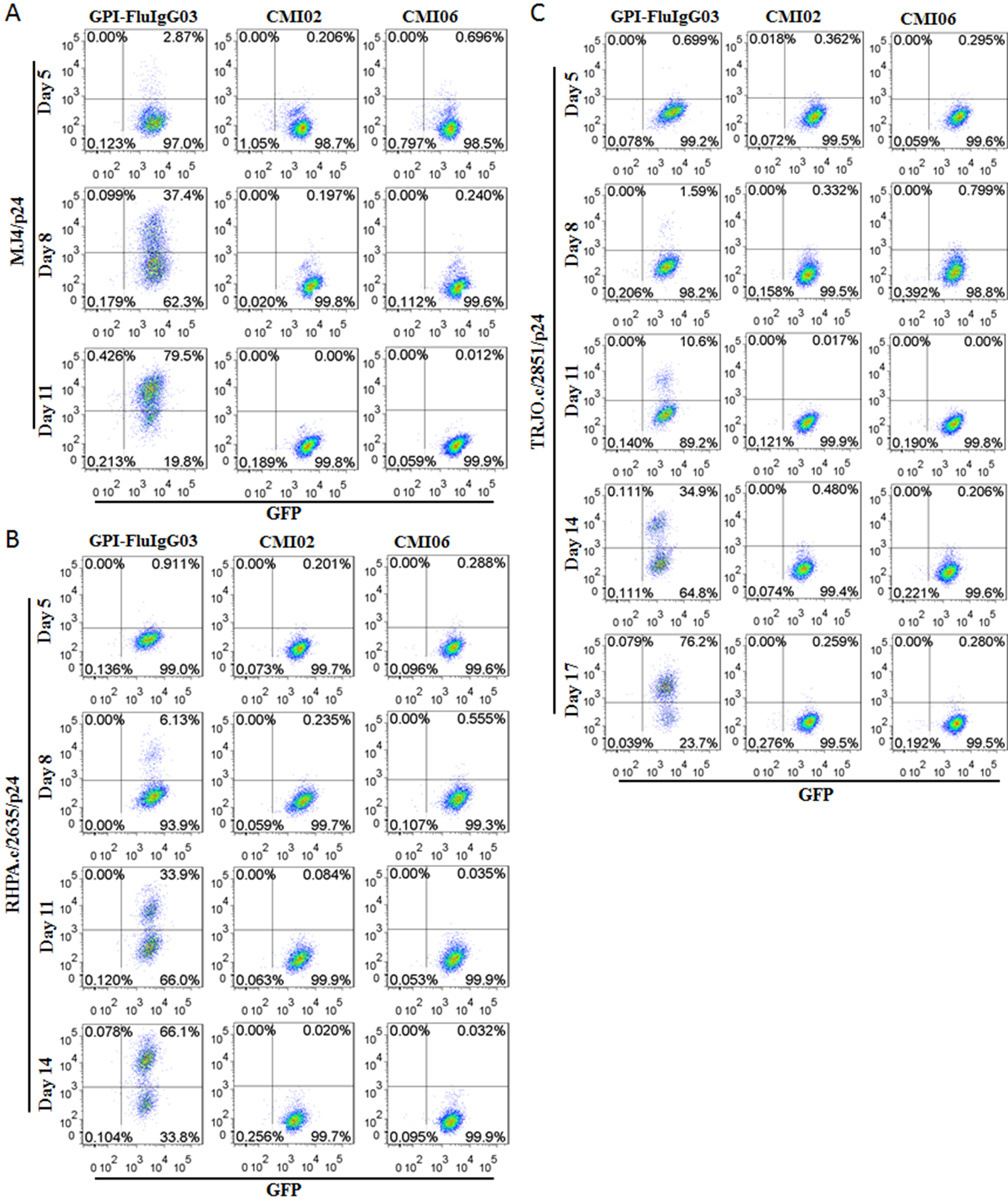

Supplement: Supplemental Material [file TEMI_A_2011616_SM4340.zip › Supplementary files/Fig. S10-R2.tif]

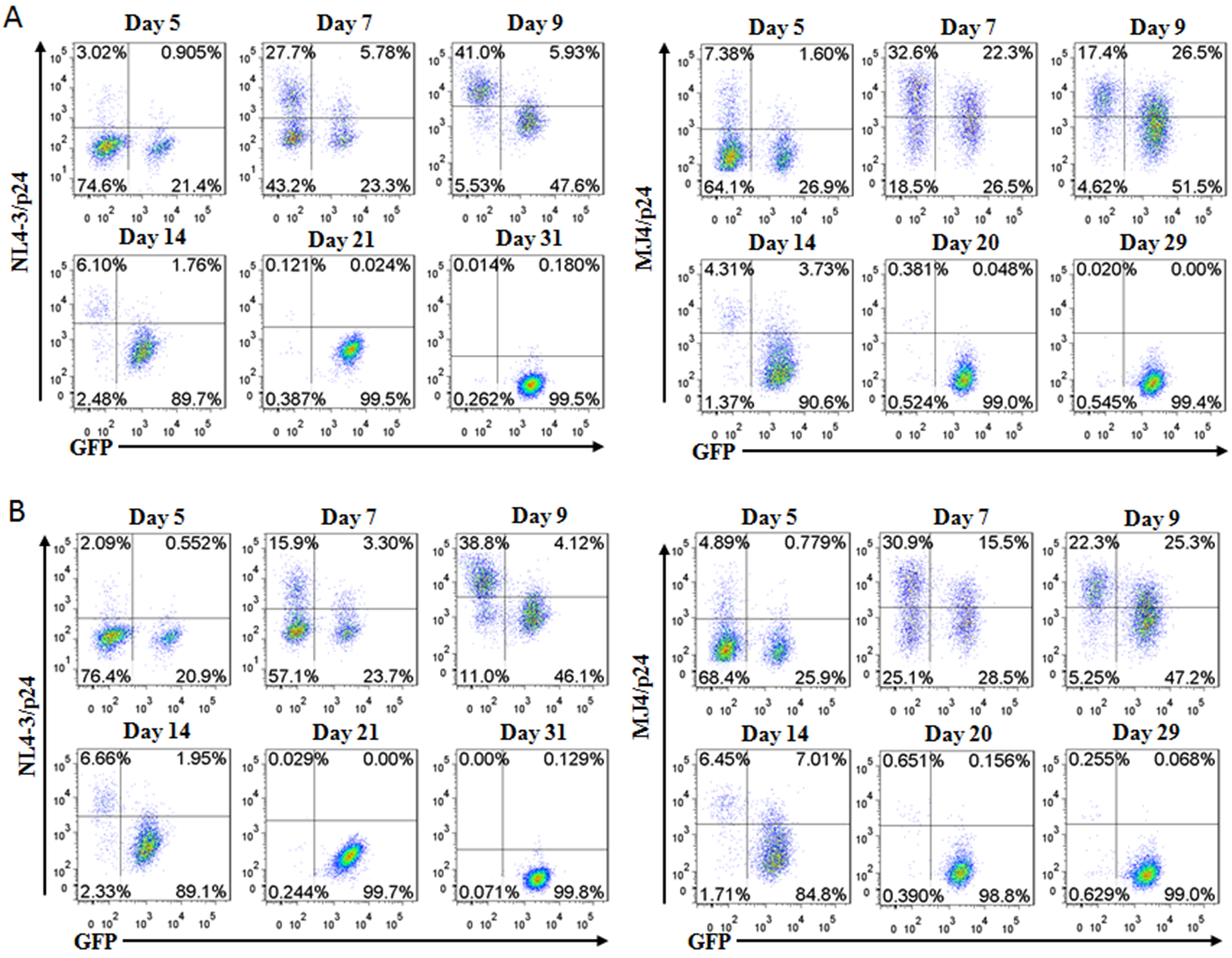

Supplement: Supplemental Material [file TEMI_A_2011616_SM4340.zip › Supplementary files/Fig. S11-R2.tif]

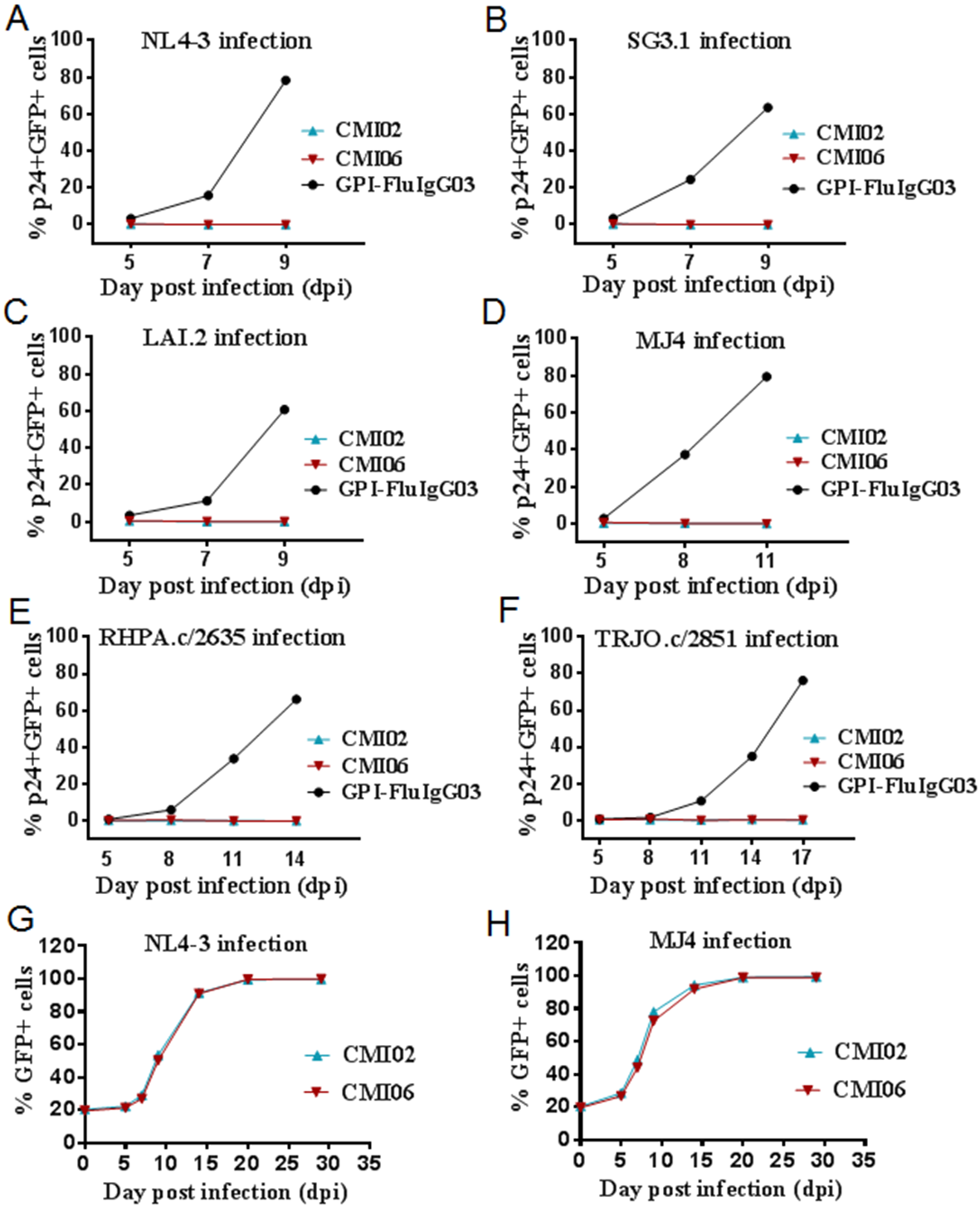

Supplement: Supplemental Material [file TEMI_A_2011616_SM4340.zip › Supplementary files/Fig. S12-R2.tif]

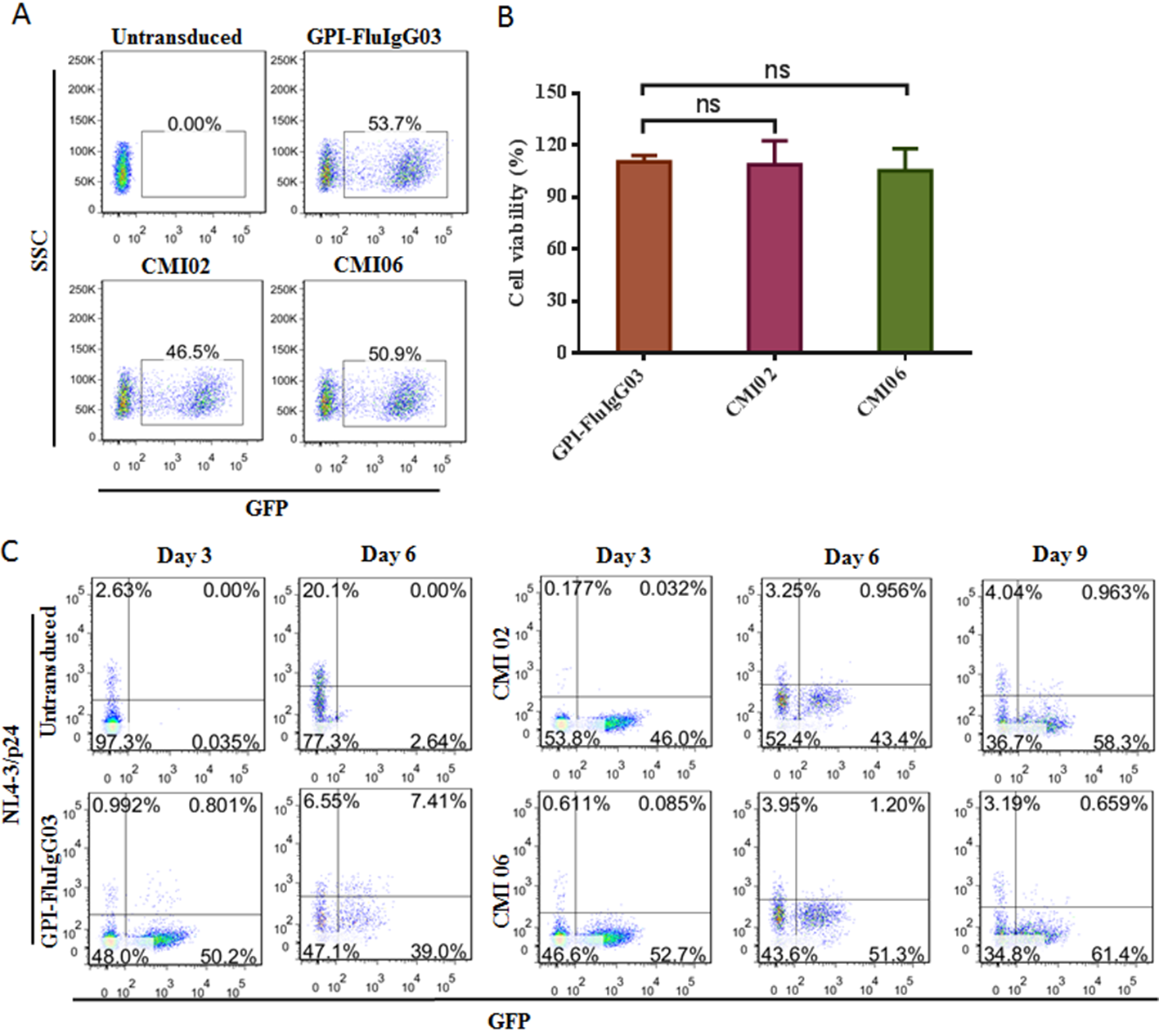

Supplement: Supplemental Material [file TEMI_A_2011616_SM4340.zip › Supplementary files/Fig. S13-R2.tif]

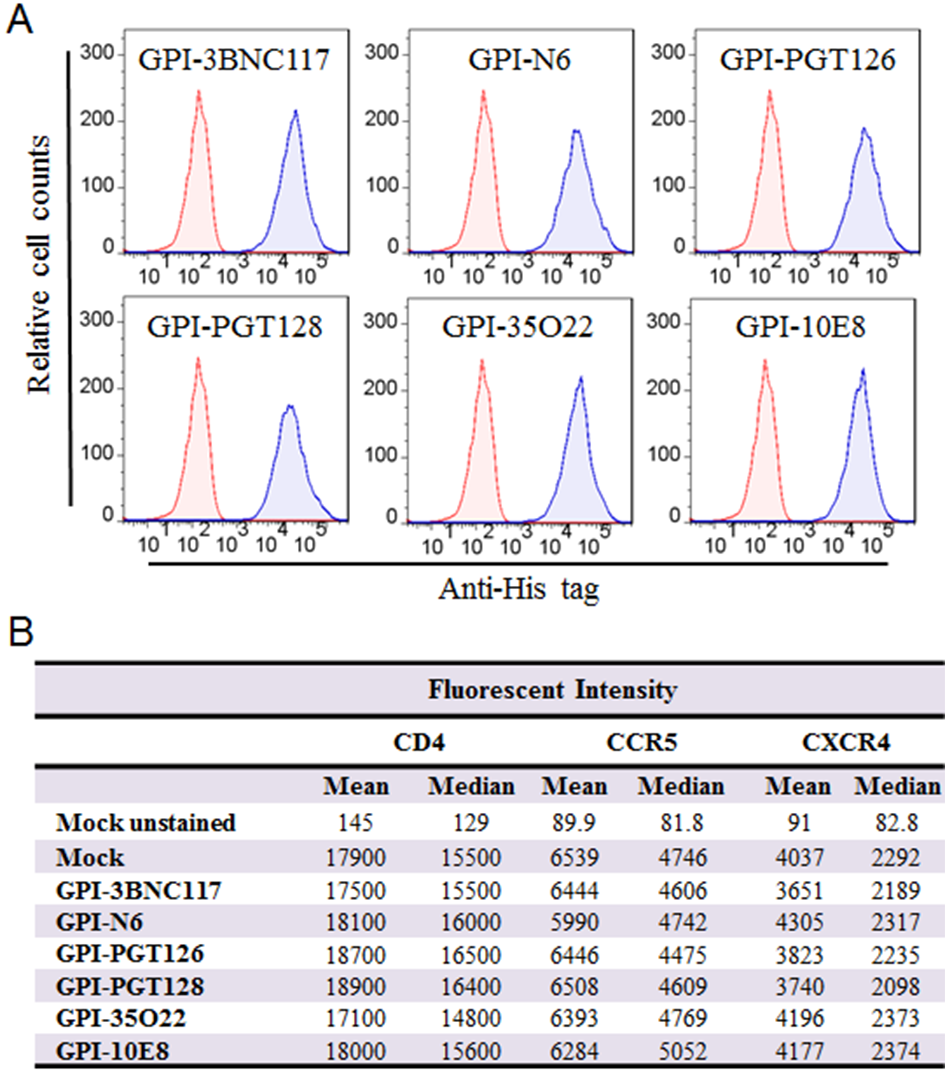

Supplement: Supplemental Material [file TEMI_A_2011616_SM4340.zip › Supplementary files/Fig. S2.tif]

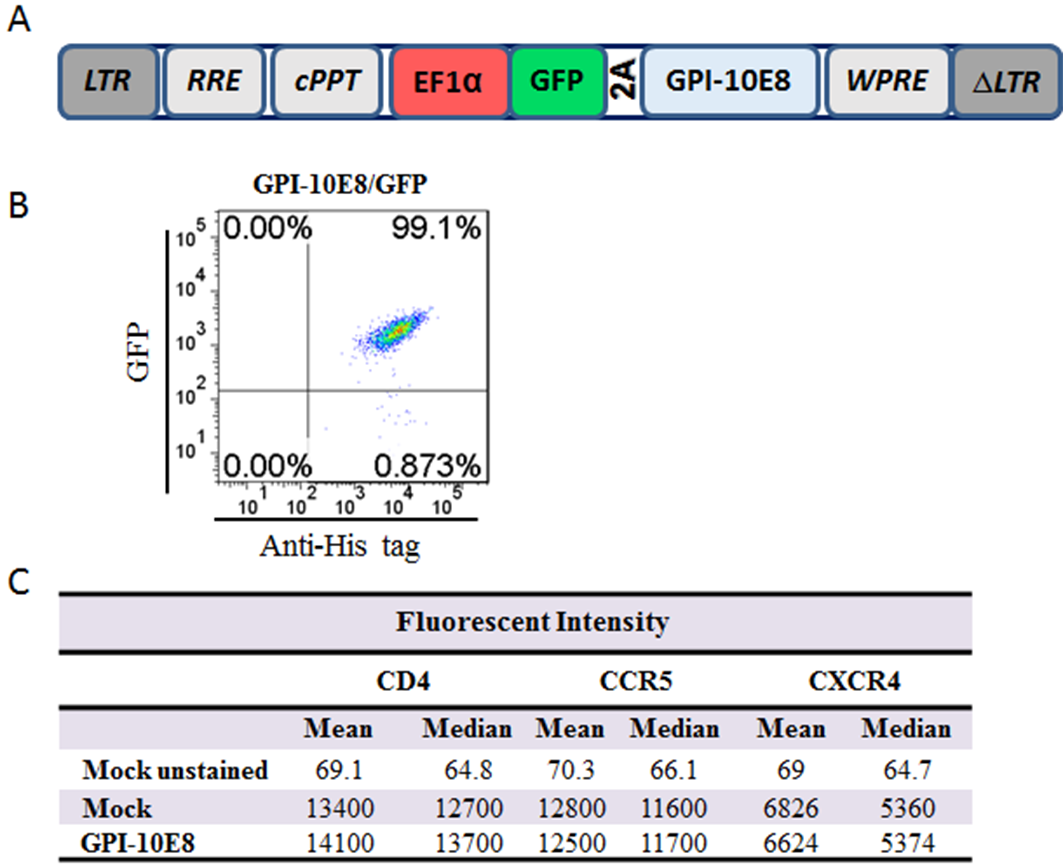

Supplement: Supplemental Material [file TEMI_A_2011616_SM4340.zip › Supplementary files/Fig. S3.tif]

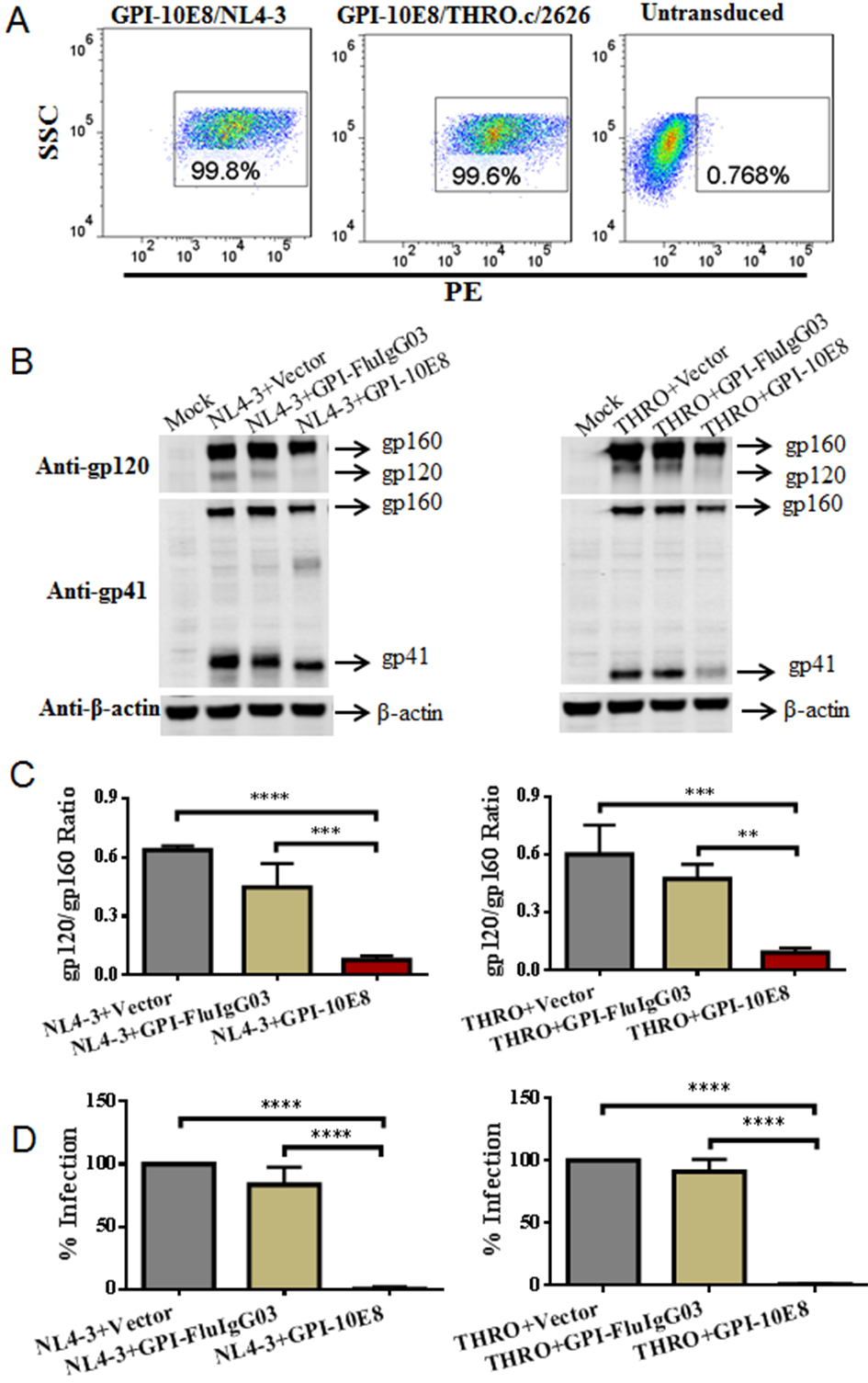

Supplement: Supplemental Material [file TEMI_A_2011616_SM4340.zip › Supplementary files/Fig. S4.tif]

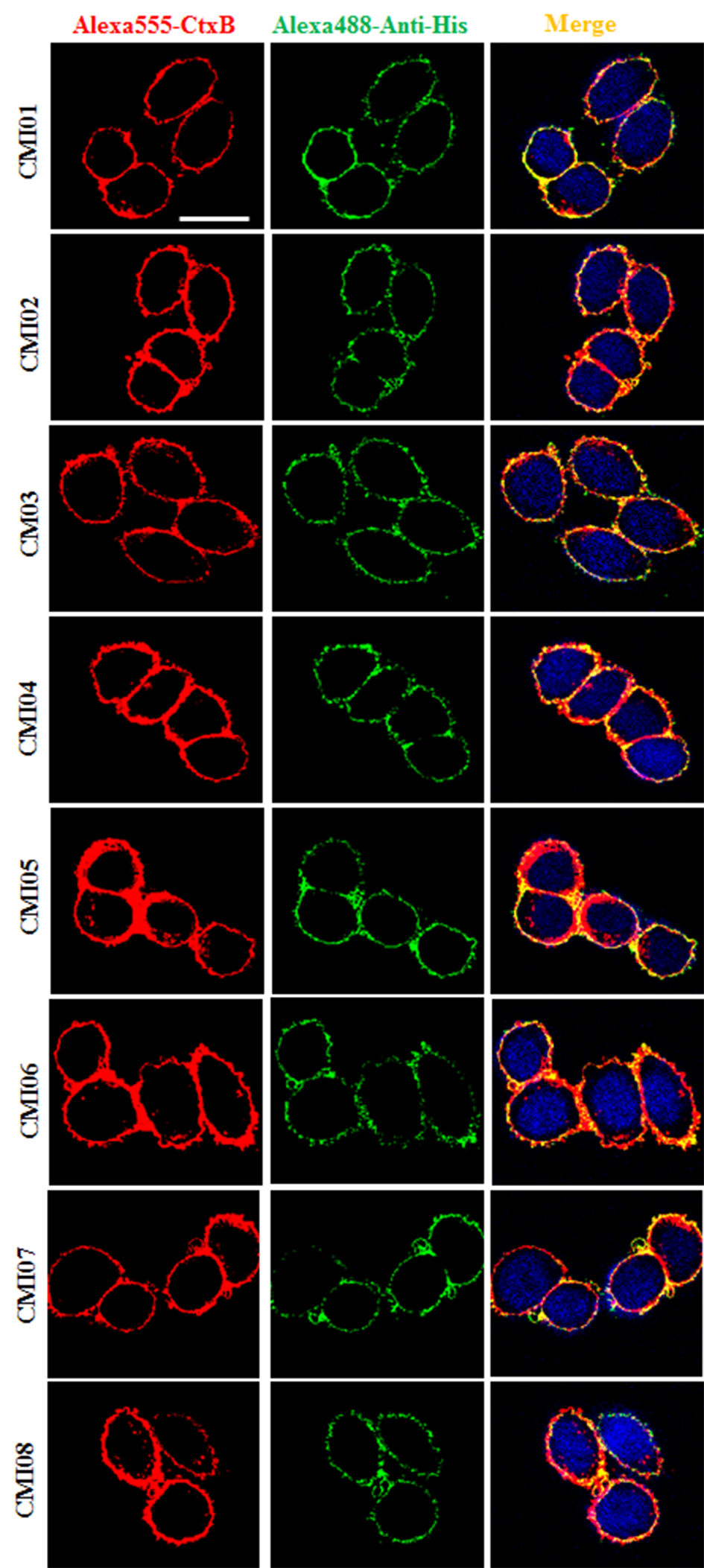

Supplement: Supplemental Material [file TEMI_A_2011616_SM4340.zip › Supplementary files/Fig. S5-R2.tif]

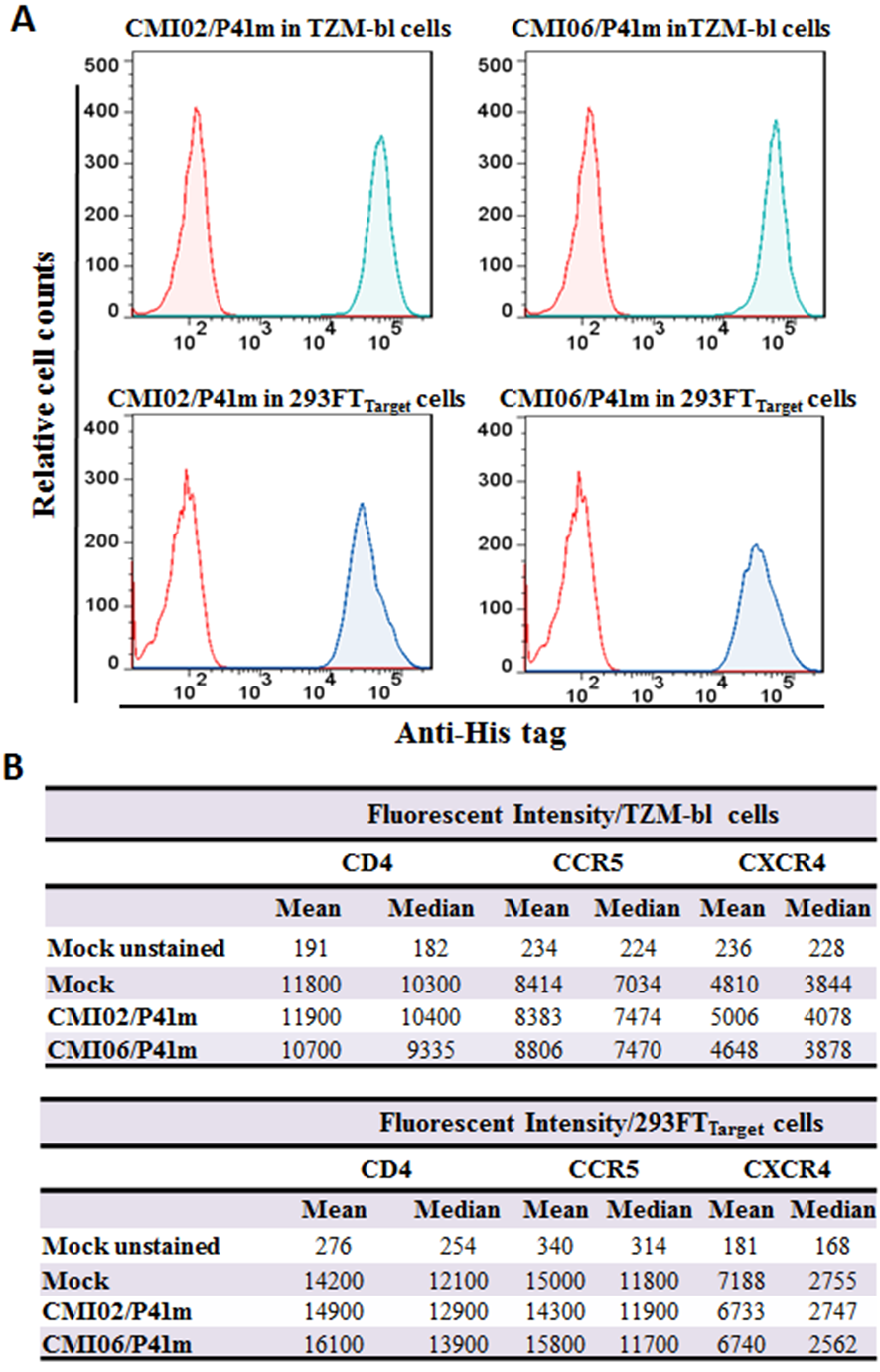

Supplement: Supplemental Material [file TEMI_A_2011616_SM4340.zip › Supplementary files/Fig. S6.tif]

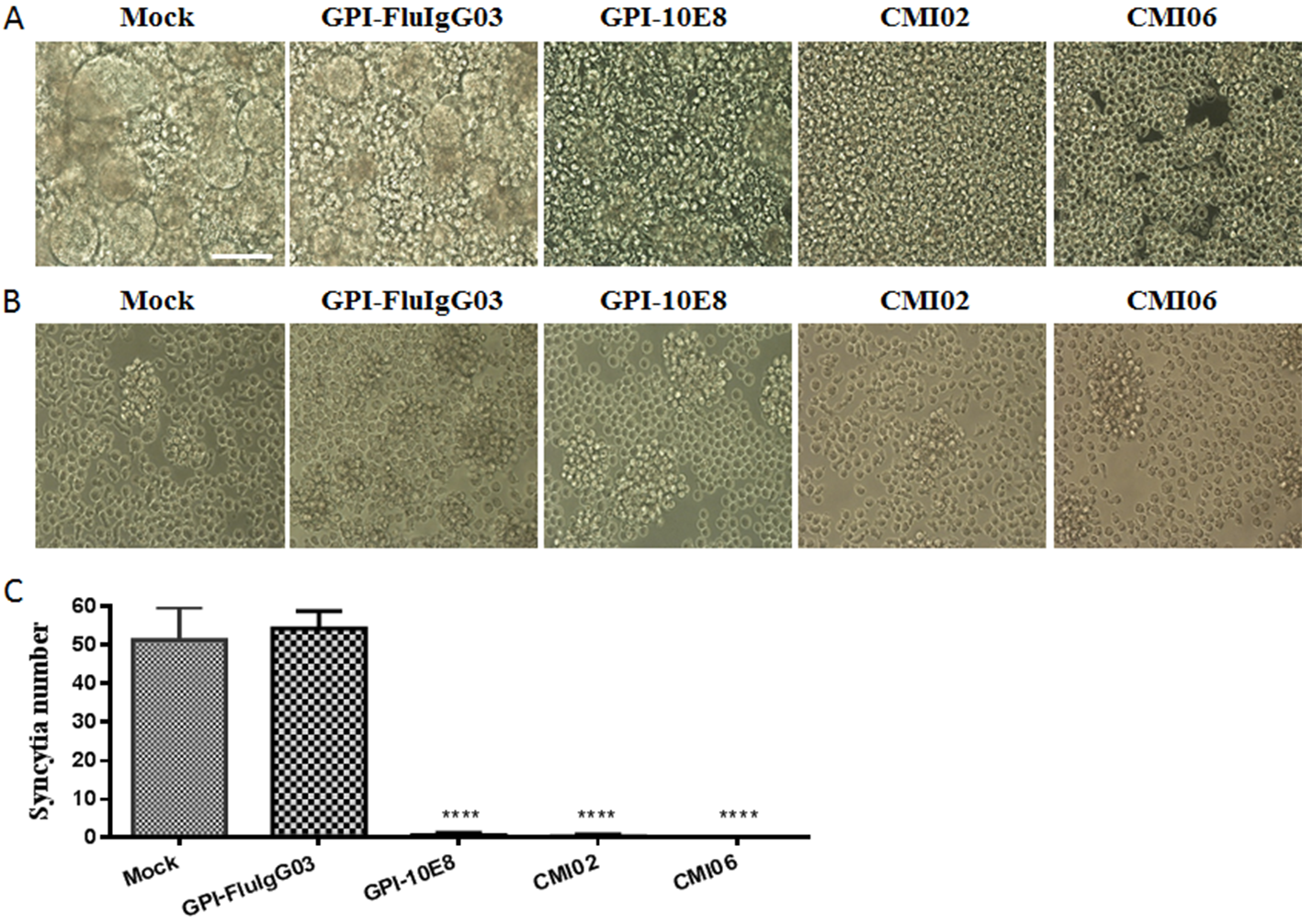

Supplement: Supplemental Material [file TEMI_A_2011616_SM4340.zip › Supplementary files/Fig. S7-R2.tif]

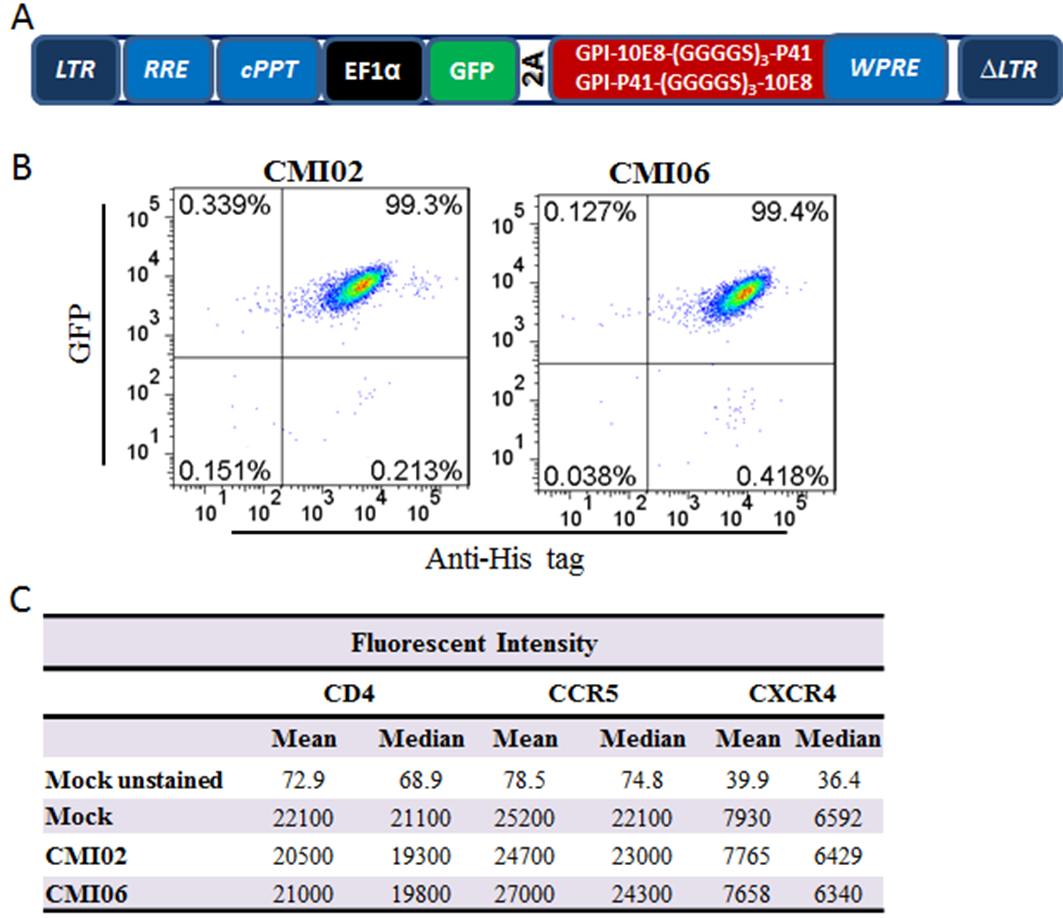

Supplement: Supplemental Material [file TEMI_A_2011616_SM4340.zip › Supplementary files/Fig. S8.tif]

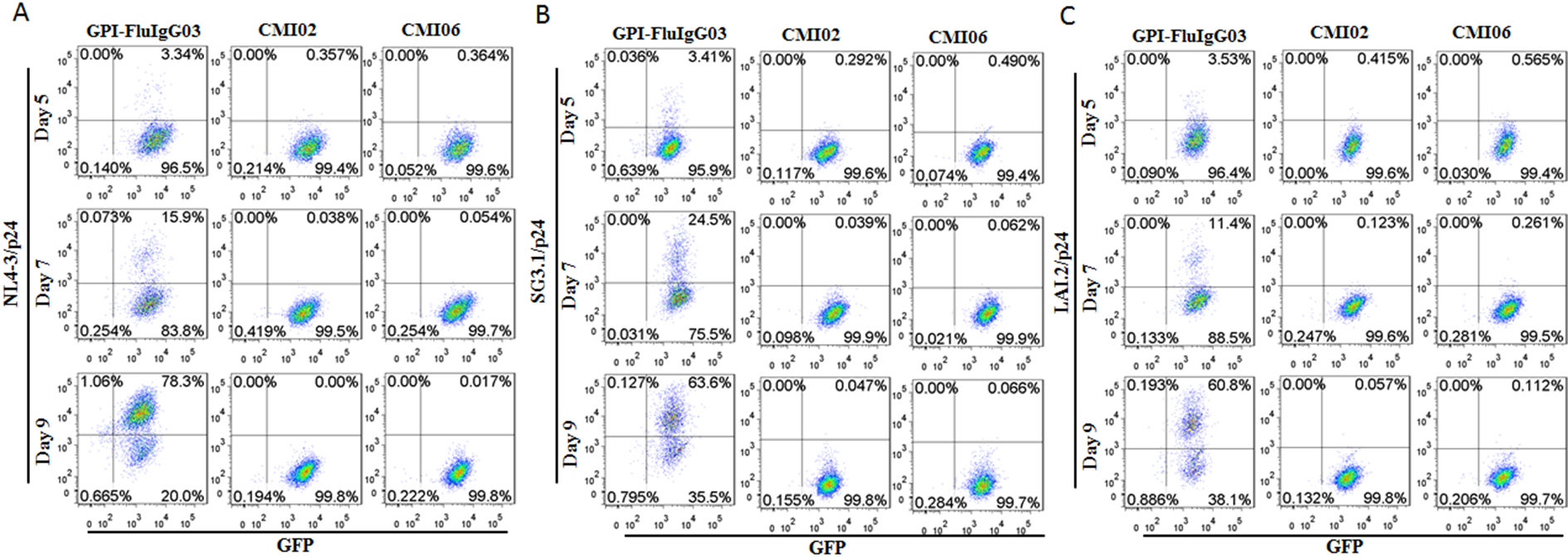

Supplement: Supplemental Material [file TEMI_A_2011616_SM4340.zip › Supplementary files/Fig. S9-R2.tif]
